# Fluorine‐Induced Lattice Oxygen Participation in 2D Layered Double Hydroxide/MXene Hybrids for Efficient Oxygen Evolution

**DOI:** 10.1002/advs.202410812

**Published:** 2024-11-11

**Authors:** Chengang Pei, Min‐Cheol Kim, Unbeom Baeck, Won Tae Hong, Jong Hun Kim, Hyungu Han, Jaekyum Kim, Sung Min Cho, Xu Yu, Jongwook Park, Ho Seok Park, Jung Kyu Kim

**Affiliations:** ^1^ School of Chemical Engineering Sungkyunkwan University (SKKU) 2066, Seobu‐Ro, Jangan‐gu Suwon 16419 Republic of Korea; ^2^ Department of Chemistry Sookmyung Women's University Seoul 04310 Republic of Korea; ^3^ School of Chemistry and Chemical Engineering Yangzhou University Yangzhou 225002 P. R. China; ^4^ Integrated Engineering Department of Chemical Engineering Kyung Hee University Gyeonggi 17104 South Korea; ^5^ SKKU Institute of Energy Science and Technology (SIEST) Sungkyunkwan University (SKKU) Suwon 16419 Republic of Korea; ^6^ SKKU Advanced Institute of Nano Technology (SAINT) Sungkyunkwan University 2066 Seobu‐ro Suwon 16419 Republic of Korea

**Keywords:** electrochemical oxygen evolution, lattice oxygen mechanism, layered double hydroxide, oxygen vacancy, surface fluorination

## Abstract

In oxygen evolution reaction (OER), the participation of lattice oxygen can break the limitation of adsorption evolution mechanism, but the activation of lattice oxygen remains a critical challenge. Herein, a surface fluorinated highly active 2D/2D FeNi layered double hydroxide/MXene (F‐LDH/MX) is demonstrated, boosting OER with the enhanced lattice‐oxygen‐mediated path. The introduction of fluorine promotes the self‐evolution of catalyst in an alkaline environment, even without an external current. It further accelerates the formation of active metal oxyhydroxides with abundant oxygen vacancies under the operating potential. The introduced oxygen vacancy activates the lattice oxygen, increasing the proportion of lattice oxygen mechanism in OER. Owing to the synergistic effects of the 2D/2D hierarchical structure and the modulated active surface, F‐LDH/MX possesses excellent electrochemical performances, including a low overpotential of 251 mV at 10 mA cm^−2^, a low Tafel slope of 40.28 mV dec^−1^, and robust stability. The water electrolyzer system with F‐LDH/MX as the anode offers the benchmark current density at a low cell voltage of 1.53 V, while the Zn‐air battery with F‐LDH/MX as the air electrode exhibits a higher power density of 75.43 mW cm^−2^. This study presents a promising strategy to design highly active electrocatalysts for energy conversion and storage.

## Graphical abstract

A 2D hybrid catalyst, consisting of MXene nanosheets coated with partially fluorinated layered double hydroxide (LDH), is successfully synthesized and exhibits excellent performance in alkaline water oxidation. Fluorine incorporation facilitates catalyst self‐reconstruction in an alkaline environment, even without external current, and enhances the formation of active metal oxyhydroxides with abundant oxygen vacancies under applied voltage.

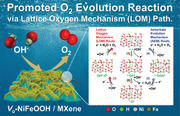

## 1 Introduction

The over‐dependence on fossil energy has exacerbated the global energy crisis, accompanied by accelerating environmental pollution, particularly greenhouse gas emissions.^[^ ^1^ ^]^ Considerable efforts have been made to develop and harness clean and efficient energy to achieve sustainable development.^[^ ^2^ ^]^ Electrochemical water splitting and metal‐air batteries are crucial parts of energy conversion and storage, however, are all hindered by the four‐electron transfer path of oxygen evolution reaction (OER) with a substantial overpotential and sluggish kinetics in an alkaline medium.^[^ ^3^ ^]^ As state‐of‐the‐art electrocatalysts in the OER, it is widely adopted using ruthenium oxide, and iridium oxide as the most active material, but their practical applications are limited by their scarcity, unaffordable cost, and poor stability.^[^ ^4^ ^]^ Hence, there has been considerable interest in developing highly active non‐precious electrocatalysts with excellent durability.^[^ ^5^ ^]^

Bimetallic catalysts, especially for the 2D layered double hydroxide (LDH), have attracted increasing interest because of the synergistic effect of multiple metal centers.^[^ ^6^ ^]^ Extensive efforts have been conducted on LDH in electrochemical water oxidation, indicating that its high performance can be assigned to the unique electronic configuration, large interlayer spacing, and large surface area exposing more active sites.^[^ ^7^ ^]^ However, the performance does not meet the needs of industrial applications because of the poor conductivity, strong tendency to aggregate, and the high‐energy barrier for the generation of active sites. The construction of heterostructures with highly conductive substrates is a promising approach that can improve the conductivity and prevent the decrease in the active area, according to previous studies.^[^ ^8^ ^]^ As an emerging family of 2D materials, MXene has been widely studied in energy conversion and storage due to its hydrophilic surface and excellent conductivity.^[^ ^9^ ^]^ Also, the abundant functional groups on its surface serve as active sites for catalytic reactions and enhance surface charge distribution, facilitating the precise integration of MXene with other nanomaterials.^[^ ^10^ ^]^ It is reported that the strong interfacial interaction owing to charge transfer between LDH and MXene can accelerate the redox process of surface metal active sites.^[^ ^11^ ^]^ Thus, LDH/MXene hybrid (LDH/MX) can be one of the most suitable candidates for improving water oxidation.

Many previously reported iron–nickel based catalysts have been commonly believed to adhere to the adsorbate evolution mechanism (AEM). However, the AEM mechanism strongly associates oxygen intermediates with catalytic activity, exhibiting a theoretical overpotential at 370 mV, which is significantly higher than the experimental overpotentials demonstrated by many published catalysts. With the discovery of the lattice oxygen mechanism (LOM), an increasing number of researchers are beginning to consider the coexistence of both AEM and LOM in most catalysts. The involvement of lattice oxygen has been proved to significantly reduce reaction barriers, thus breaking free from the 370 mV limitation. Unfortunately, the LOM involves severe surface reconstruction with the participation of lattice oxide, leading to low stability. Thus, it is crucial to realize an OER catalyst that can enhance the contribution of the LOM mechanism without compromising electrode stability.

Herein, we enhance the LOM capability of LDH/MX catalysts for OER through surface fluorination while maintaining their catalytic stability. The 2D/2D hybrid architecture of partially fluorinated LDH/MX (F‐LDH/MX) offers a robust structure with sufficient active surface area and accelerates ion diffusion. The F‐LDH/MX electrode shows an OER performance of a low overpotential of 251 mV at a current density of 10 mA cm^−2^ with a low Tafel slope of 40.28 mV dec^−1^ in alkaline electrolytes. We demonstrate that partially F‐treated LDH on MXene can spontaneously convert into hydroxides in an alkaline solution without an applied potential. Under an electric current, the metallic species further transform into high‐valence oxyhydroxides. Experiments and theoretical calculations proved that abundant oxygen vacancies in FeNiOOH improve the contribution of LOM in OER. Regardless of the enhanced LOM, the F‐LDH/MX electrode maintains the electrode stability compared with LDH/MX electrodes. We also demonstrate the realistic application of F‐LDH/MX in water electrolysis cells and zinc‐air batteries. This work provides significant guidelines for designing highly efficient and stable hybrid nanomaterials based on MXene in energy conversion and storage.

## 2 Results and Discussion

F‐LDH/MX nanohybrids were fabricated by partially fluorinating FeNi‐LDH/Ti_3_C_2_ MXene nanosheets. First, multi‐layer Ti_3_C_2_ MXene with an accordion‐like structure was fabricated via the HF etching of Ti_3_AlC_2_. Then the few‐layer Ti_3_C_2_ MXene was exfoliated by ultrasonic treatment in dimethyl sulfoxide for several hours under flowing Ar gas, avoiding further oxidation. The LDH/MX nanohybrids were prepared by co‐precipitating Fe^3+^ and Ni^2+^ in the presence of few‐layer Ti_3_C_2_ MXene under reflux with nitrogen gas as the protecting atmosphere (Figure S1, Supporting Information). Subsequently, the prepared nanohybrids were fluorinated by HF generated at 150°C in flowing Ar. The generation of HF gas can be adequately adjusted by controlling the dosage of the precursor ammonium fluoride. In this method, only the surface of the material is fluorinated, while the nanosheet morphology is preserved completely, which may expose more active sites.^[^ ^12^ ^]^

**Figure 1:**
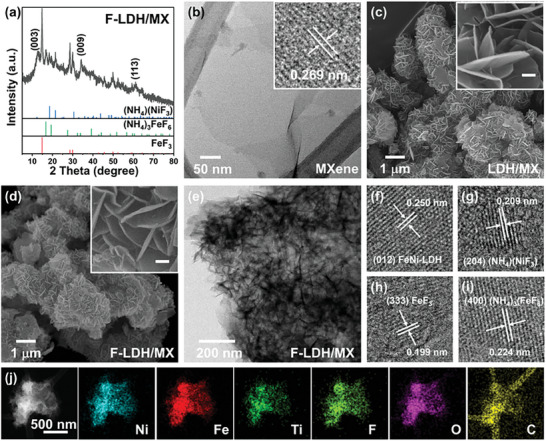
a) XRD patterns of the F‐LDH/MX sample. b) TEM image of 2D MXene (Inset: HRTEM image of MXene). SEM images of c) LDH/MX and d) F‐LDH/MX (Inset: the scale bar is 100nm). e) TEM and f–i) HRTEM images of F‐LDH/MX. j) STEM image and corresponding elemental mappings of F‐LDH/MX (The scale bar is 500 nm).

X‐ray diffraction (XRD) was employed to characterize the change in crystal structure from LDH to F‐LDH/MX catalysts. The typical characteristic XRD peaks for LDH precursors are demonstrated (Figure S2, Supporting Information). Compared to pristine LDH, no other peaks of Ti_3_C_2_ MXene were found in LDH/MX arising from the low content or low crystallinity of the few‐layer MXene, which is consistent with other studies.^[^ ^13^ ^]^ After low‐temperature fluorination, the strongest characteristic peaks of F‐LDH/MX at 12.64°, 34.25°, and 61.19° correspond to FeNi‐LDH, indicating that the partial fluorination occurred on the surface of LDH/MX, maintaining most of the bulk LDH/MX structure. New characteristic peaks at 14.84°, 28.63°, 29.91°, 45.76°, 50.00°, 52.26°, 59.49°, and 64.30° were indexed as the cubic FeF_3_ (PDF No. 84–1101). Other additional peaks correspond to the diffraction peaks of (NH_4_)(NiF_3_) (PDF card No. 86–1837) and (NH_4_)_3_FeF_6_ (PDF card No. 77–0147) because of low‐temperature fluorination (**Figure** **1**a).

Transmission electron microscopy (TEM) images were taken to visualize details of the sample microstructure and surface morphology. The as‐prepared few‐layer Ti_3_C_2_ nanosheets showed flat surfaces via TEM, suggesting the successful etching of the Ti_3_AlC_2_ phase and delamination of the accordion‐like multilayer Ti_3_C_2_. High‐resolution TEM (HRTEM) measurement indicated the crystalline nature of MXene nanosheets by clear lattice fringes of 0.269 nm, corresponding to the ($0\bar{1}10$) plane of Ti_3_C_2_ MXene (Figure 1b).^[^ ^10a^ ^]^ The Raman spectrum also confirmed the successful fabrication of few‐layer MXene (Figure S3, Supporting Information). Two peaks at 201 and 621 cm^−1^ corresponded to A_1g_ vibrations of Ti and C atoms, while another peak at 400 cm^−1^ belonged to E_g_ vibrations of Ti, C, and surface functional groups. Additionally, the two peaks at 1330 and 1551 cm^−1^ contributed to the D‐band and G‐band of carbon, respectively. Fe and Ni ions can be adsorbed and anchored on the MXene surface owing to abundant ─OH and ─F groups on the surface of MXene nanosheets.^[^ ^13^, ^14^ ^]^ The scanning electron microscopy (SEM) image revealed that after in situ co‐precipitation, NiFe‐LDH nanosheets with a thickness of ≈20 nm were grown vertically on the surface of MXene (Figure 1c). Elemental mapping analysis with a uniform distribution confirmed the presence of Fe, Ni, Ti, C, and O in LDH/MX (Figure S4, Supporting Information). Although pristine LDH has a layered structure (Figure S5, Supporting Information), the 2D/2D structure of LDH/MX revealed unique advantages in preventing agglomeration and exposing more active sites. No obvious morphology transformation can be found after low‐temperature fluorination, suggesting the preservation of the structural advantages (Figure 1d). The morphology of F‐LDH/MX was further analyzed with TEM. Figure 1e shows the 3D interconnected architecture with a defined outline, in which the partially fluorinated LDH nanosheets adhere to 2D MXene. Well‐resolved lattice fringes of 0.250, 0.209, 0.199, and 0.224 nm were shown in HRTEM, which were in good agreement with the (012) plane of FeNi‐LDH, (204) plane of (NH_4_)(NiF_3_), (333) plane of FeF_3_, and (400) plane of (NH_4_)_3_(FeF_6_), respectively (Figure 1f–i). The elemental mapping images indicated the uniform distribution of Ni, Fe, Ti, F, O, and C demonstrating the fluorination of the metal hydroxide surface and retention of the MXene structure (Figure 1j). The unique edge‐to‐face 2D/2D architecture of F‐LDH/MX is indispensable during the electrocatalytic process because it is desirable to expose more edges, provide abundant pathways for ion diffusion and facilitate charge transfer and mass diffusion during the electrochemical reactions.^[^ ^15^ ^]^

**Figure 2:**
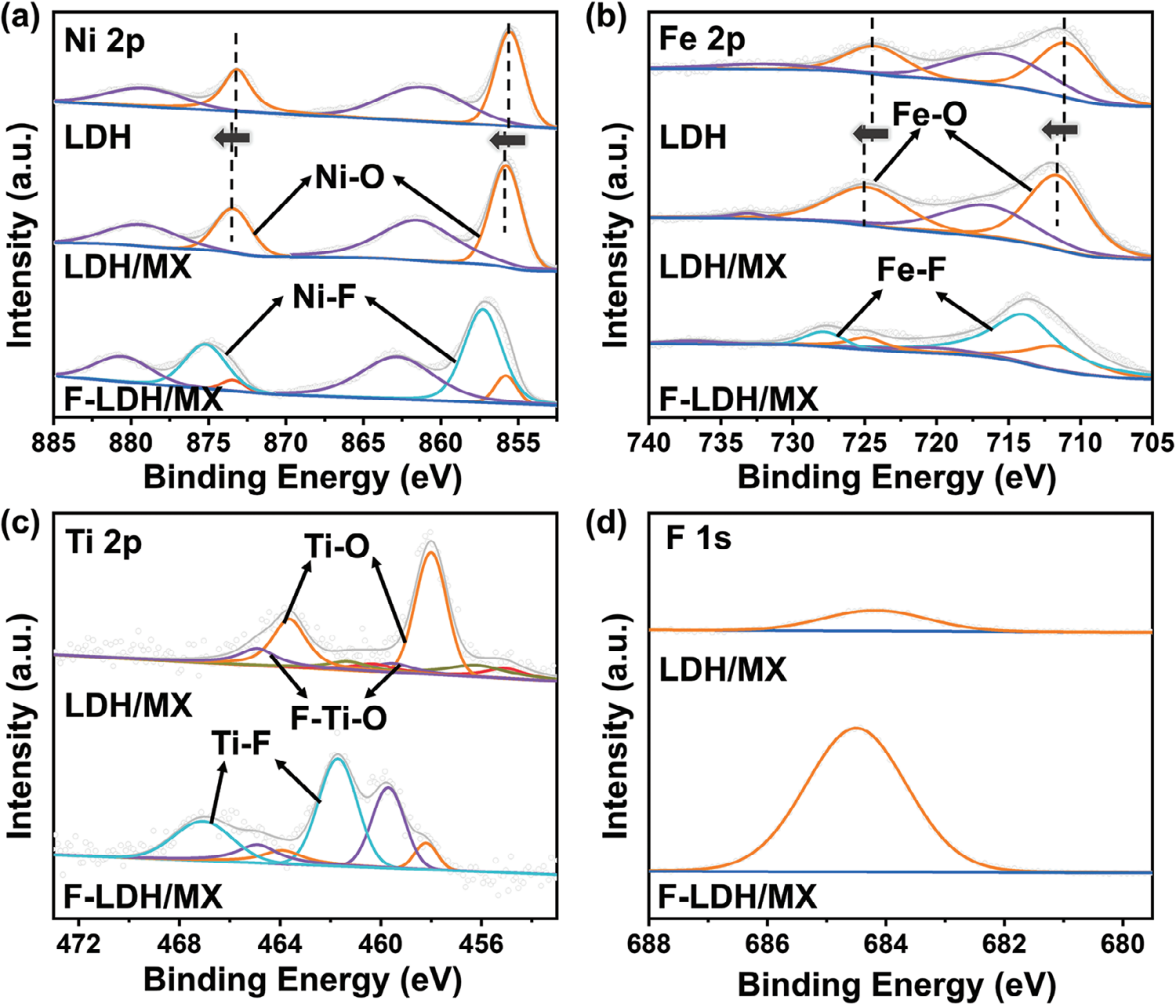
a–d) High‐resolution‐fitted XPS spectra of Ni 2p, Fe 2p, Ti 2p, and F 1s for LDH, LDH/MX, and F‐LDH/MX.

X‐ray photoelectron spectroscopy (XPS) was conducted on the related catalysts to analyze the surface chemical state. All high‐resolution spectra were calibrated to the C 1s peak at 284.80 eV. In the spectrum of Ni 2p, two components (855.48 eV for 2p_3/2_ and 872.98 eV for 2p_1/2_) were observed for LDH and each component was deconvoluted into peaks as Ni–O accompanied by satellite peaks (**Figure** **2**a). Compared to pristine LDH, a slight shift of the peaks was found on LDH/MX demonstrating the strong interaction and charge transfer between LDH and MXene nanosheets, which is consistent with other studies.^[^ ^11b^ ^]^ After the in situ fluorination process, electrons are withdrawn easily from metallic elements because of the high electronegativity of F. Thus, two peaks located at 857.33 and 875.25 eV were found, which were assigned to Ni─F bond with much higher binding energy compared to Ni─O. A similar result was found on Fe (Figure 2b). Because of the strong electron‐withdrawing effect of MXene and surface fluorination treatment, the XPS peaks of Fe shifted to the higher binding energy region, which is considered to be favorable for the formation of electrochemically active sites in OER.^[^ ^16^ ^]^ The chemical states of Ti in MXene, LDH/MX, and F‐LDH/MX were detected as well. In the pristine few‐layer MXene nanosheet, the spectrum of Ti 2p can be divided into Ti─C (455.05 eV and 460.06 eV), Ti^2+^/Ti^3+^ (456.09 and 461.24 eV), Ti─O (458.13 and 462.76 eV), and F─Ti─O (459.56 and 464.70 eV), respectively (Figure S6, Supporting Information). However, no obvious peaks of Ti─C and Ti^2+^/Ti^3+^ were found in LDH/MX because of the low stability of the surface sites of the MXene nanosheet, even though the preparation process was performed under flowing N_2_ gas (Figure 2c).^[^ ^13^, ^14^, ^17^ ^]^ F‐LDH/MX showed a pair of unexpected peaks at 461.63 and 467.01 eV, corresponding to the Ti─F bond because of the low‐temperature fluorination treatment. In the F 1s spectra, an obvious shift of F was observed between LDH/MX and F‐LDH/MX from 683.9 to 684.4 eV, indicating the successful formation of F‐Metal (Ni or Fe) bonding (Figure 2d). Also, the content of F in F‐LDH/MX increased dramatically to nearly 19 times that in LDH/MX which came from F‐doping in the HF‐etching process (Table S1, Supporting Information). Considering the XPS analysis, it is apparent that the surface fluorination has a significant effect on the valence state of metal elements, and the resulting higher binding energy peaks of metal sites are beneficial for the generation of actual OER active sites and the adsorption of reaction intermediates on the surface.^[^ ^16^ ^]^

**Figure 3:**
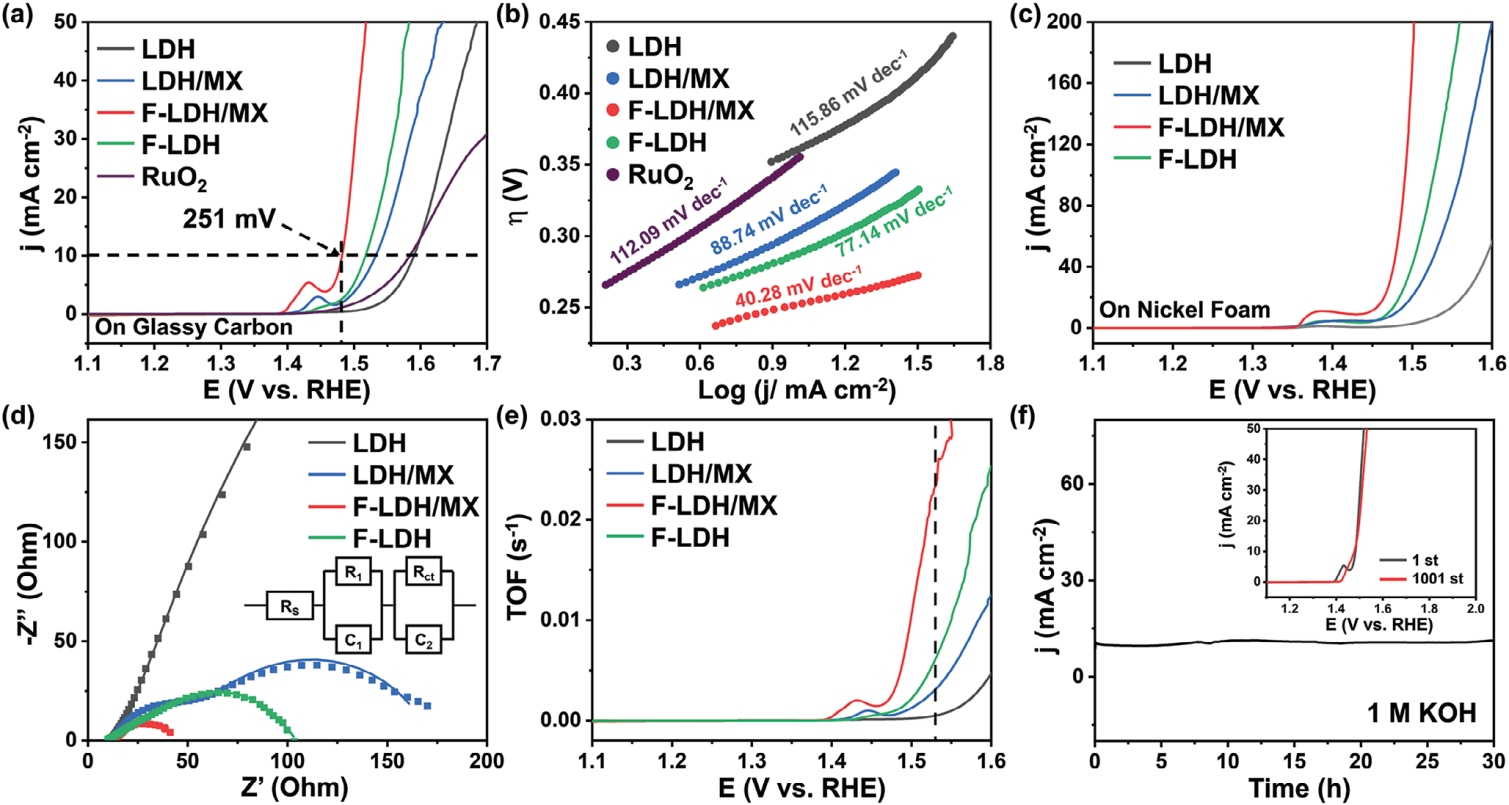
a) Polarization curves of LDH, LDH/MX, F‐LDH/MX, F‐LDH, and RuO_2_ samples loaded on a glassy carbon electrode (0.07 cm^−2^) in 1m KOH solution at a scan rate of 5mV s^−1^. The dashed line corresponds to the potential at 10 mA cm^−2^ for each catalyst. b) Tafel plots of these samples. c) Polarization curves of LDH, LDH/MX, F‐LDH/MX, and F‐LDH loaded on NF in 1 m KOH at a scan rate of 2 mV s^−1^. d) Nyquist plots of LDH, LDH/MX, F‐LDH/MX and F‐LDH samples. (Inset: The equivalent circuit model for electrochemical impedance tests). e) TOF value of these samples. The dashed line corresponds to the TOF value at 300 mV for each catalyst. f) Chronopotentiometry curves of the F‐LDH/MX at a current density of 10 mA cm^−2^ (Inset: Stability test for F‐LDH/MX by CV scanning before and after 1000 cycles).

A series of electrocatalysts dropped on inert glass carbon electrodes, including MXene, LDH, LDH/MX, F‐LDH/MX, partially fluorinated LDH (F‐LDH), and RuO_2_, were measured for the electrochemical OER performance in an alkaline solution. The main material F‐LDH/MX exhibited a strong redox peak at ≈1.43V, proving the valence state of surface Ni changing from Ni^2+^ to Ni^3+/4+^ from NiFeOOH, which is identified as the real active site in catalyzing oxygen evolution.^[^ ^11b^ ^]^ It was reported that the charge transfer between LDH and MXene can accelerate the redox process of surface metal active sites,^[^ ^11^ ^]^ which is consistent with our XPS results and reasonably explains why LDH/MX exhibits an apparent oxidation peak compared with pristine LDH. A redox peak around 1.46 V was also found in F‐LDH. After low‐temperature fluorination, the metal atomic sites on the LDH surface are activated since the introduced F deprived the electrons from the metal. It has been reported that such electron‐hungry metal species can easily convert to highly active metal oxyhydroxide.^[^ ^18^ ^]^ The potential at 10 mA cm^−2^_geo_, which is approximately the minimum current density to achieve a solar‐to‐fuel conversion efficiency of 10% for artificial photosynthesis devices, is also a significant parameter for measuring the OER performance.^[^ ^19^ ^]^ No obvious OER performance was found on MXene (Figure S7, Supporting Information), demonstrating that the role of MXene is to act as a conductive carrier responsible for charge transfer in the bulk phase and regulating the electronic structure of surface metal (Fe and Ni) sites rather than the catalytic active site for the OER. Pristine LDH exhibited the next‐lowest performance for OER after pristine MXene, with an overpotential of 360 mV, which is far from the needs for industrial applications (**Figure** **3**a). The performance was improved after loading 2D conductive MXene nanosheets. F‐LDH/MX exhibited the lowest overpotential among the examined catalysts. In detail, a low overpotential of 251 mV was needed to reach 10 mA cm^−2^, which was significantly lower than LDH/MX (302 mV), F‐LDH (286 mV), and the classical OER benchmark catalyst RuO_2_ (352 mV). The Tafel slope, as another critical indicator for OER, was measured to gain insight into the reaction kinetics of the catalysts (Figure 3b). Among the catalysts, F‐LDH/MX exhibited the lowest Tafel slope of 40.28 mV dec^−1^, which was significantly lower than LDH (115.86 mV dec^−1^), LDH/MX (88.74 mV dec^−1^), F‐LDH (77.14 mV dec^−1^), and RuO_2_ (112.09 mV dec^−1^), indicating the most facile reaction kinetics among the examined catalysts. In addition, the OER performances of these samples on nickel foam (NF) were also studied (Figure 3c; Figures S8 and S9, Supporting Information). Compared with those on glassy carbon (GC), both the overpotential and the Tafel slope of these samples exhibited significant improvement with the same trend in catalytic performance. It may be attributed to the high conductivity and large surface area of NF compared with GC.^[^ ^20^ ^]^ The interfacial properties of the catalyst‐modified electrode were further investigated by electrochemical impedance spectroscopy (EIS) to gain deeper insight into the mechanism of these catalysts. Figure 3d shows the Nyquist plots and the corresponding equivalent circuit model, in which an uncompensated solution resistance (R_s_) was connected to two parallel combinations of a capacitance (C_1_ and C_2_) and a resistor (R_1_ and R_ct_) in series (Figure 3d). Among them, R_1_ at high frequencies was related to the dielectric properties and the resistivity of the in situ formed oxide film in oxygen evolution reaction while R_ct_ at low frequencies showed the resistance of charge transfer between the interface of the electrolyte and electrode.^[^ ^21^ ^]^ The smallest R_ct_ value was also observed on the F‐LDH/MX electrode of 24.97 Ω, which is much smaller than other catalysts, including LDH (1157.00 Ω), LDH/MX (112.50 Ω), and F‐LDH (48.77 Ω), demonstrating its rapid charge transfer kinetics (Table S2, Supporting Information). Both the results of Tafel slope and EIS indicate the fast reaction kinetics of F‐LDH/MX in catalyzing oxygen evolution.

The specific activity and turnover frequency (TOF) were further introduced to rationally investigate the intrinsic activity of the catalysts. The specific activity denotes the activity per unit surface area of the catalyst. This can be obtained by normalizing the current over the electrochemical surface area (ECSA). The double‐layer capacitance (C_dl_) of these catalysts was first measured by cyclic voltammetry (CV) at different scan rates to obtain ECSA (Figure S10, Supporting Information). Among these catalysts, the largest values of C_dl_ and ECSA were observed on F‐LDH/MX, which were assigned to the synergistic effects of HF etching and the construction of a hierarchical structure. Figure S10f (Supporting Information) shows the specific activity of these catalysts. It is apparent that F‐LDH/MX showed the highest performance per unit surface area. At an overpotential of 300 mV, the current density of F‐LDH/MX was 59.96 mA cm^−2^, which is ≈31.75, 5.59, and 3.04 times higher than LDH, LDH/MX, and F‐LDH, respectively. The TOF is another important parameter for explaining the intrinsic properties of an electrocatalyst. According to previous studies, MXene is OER‐inactive with negligible activity,^[^ ^11b^ ^]^ which is also consistent with our electrochemical (EC) polarization test results on the pristine MXene. The catalytic active site is controversial for NiFe‐LDH as there are reports on both Ni and Fe regarded as the OER active site.^[^ ^22^ ^]^ Thus, we consider both Fe and Ni as the active site in all mentioned catalysts. Figure 3e presents the TOF curves of the catalysts, and F‐LDH/MX exhibited the best performance. In particular, the value of F‐LDH/MX at an overpotential of 300 mV was 0.24 s^−1^, which is significantly higher than LDH (0.005 s^−1^), LDH/MX (0.003 s^−1^), and F‐LDH (0.006 s^−1^). In addition, this value is remarkable compared to other transition metal‐based catalysts (0.04–0.21 s^−1^).^[^ ^23^ ^]^ The catalytic performance, including the overpotential at 10 mA cm^−2^ and Tafel slope, was compared with other MXene‐based OER catalysts in alkaline media (Table S3, Supporting Information). Also, the performance was superior to most state‐of‐the‐art FeNi catalysts with the same substrate (Table S4, Supporting Information).

The long‐term stability is another essential indicator to evaluate the electrocatalyst performance, which cannot be ignored for real applications. The consecutive CV was first employed to measure the dynamic stability of samples (Figure 3f). The polarization curves of F‐LDH/MX before and after 1000 CVs almost overlapped, particularly when the current density was below 20 mA cm^−1^, indicating excellent cyclability as an anode material in an alkaline medium. Long‐term chronoamperometry (CA) measurements were also measured to determine the stability of catalysts. After 30 h, no significant decrease in current density was found. The stability of pristine MXene was also studied in detail (Figure S7, Supporting Information). The OER performance of MXene decreased significantly in the first ten cycles, while no further decline was found from the 11th cycle even after 1018 cycles. It may attribute from the surface oxidation of Ti sites and the aggregation of nanosheets. These results show that the F‐LDH protects the inner MXene from oxidation and the unique 2D/2D structure greatly reduced the aggregation.

**Figure 4:**
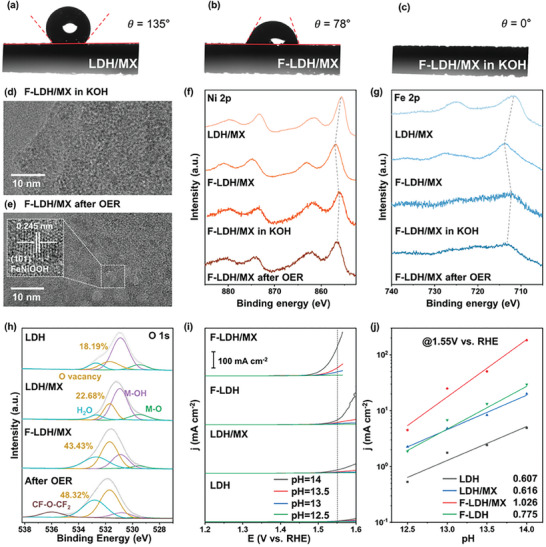
(a) The contact angle measurement of a) LDH/MX, b) F‐LDH/MX, and c) F‐LDH/MX immersed in 1 m KOH. The TEM images of d) F‐LDH/MX in KOH and e) F‐LDH/MX after OER. The high‐resolution spectra of f) Ni 2p and g) Fe 2p for LDH/MX, F‐LDH/MX, F‐LDH/MX in KOH, and F‐LDH/MX after OER. h) The high‐resolution spectra of O 1s for LDH, LDH/MX, F‐LDH/MX, and F‐LDH/MX after OER. i) OER polarization curves of LDH, LDH/MX, F‐LDH, and F‐LDH/MX in KOH electrolyte at 5 mV s^−1^ from pH 12.5 to 14 on the RHE scale. j) OER specific activities of all catalysts at 1.55 V versus different pHs of KOH.

To better understand the reaction mechanism and the real OER active sites, we made a detailed comparison of catalysts before and after the reaction including the contact angle, TEM, Raman, and XPS. We measured the wettability of the electrode surfaces with contact angle measurement, which gives an idea on the gas adsorption/desorption behaviors and the kinetics of the electrochemical reaction (**Figure** **4**a–c). Despite the well‐known hydrophilicity of MXene with reported contact angles in the 30–80° range,^[^ ^24^ ^]^ LDH/MX on NF exhibited hydrophobicity with a relatively large contact angle of 135° because of the high mass loading of LDH. The surface hydrophobicity significantly decreases after partial fluorination, with the contact angle decreasing to 78°. Unexpectedly, the surface of F‐LDH/MX became super‐hydrophilic when it was immersed in 1 m KOH without any applied potential. Such super‐hydrophilicity not only accelerates the nucleophilic adsorption of water molecules improving the OER kinetics,^[^ ^25^ ^]^ but also facilitates improved charge transfer capabilities between the electrode surface and the electrolyte.^[^ ^15^ ^]^ In addition, it was reported that a super‐hydrophilic surface can be beneficial to the diffusion of gas bubbles especially in high current density.^[^ ^26^ ^]^ Furthermore, the change in the contact angle of F‐LDH/MX with the introduction of alkaline electrolytes indirectly indicates the surface reconstruction of F‐LDH/MX under alkaline solution. TEM images reveal the nanosheet structure of F‐LDH/MX both in KOH and after OER (Figure 4d,e; Figure S11, Supporting Information). The phase of metal fluoride disappeared when F‐LDH/MX was immersed in KOH, and it showed the amorphous phase without an obvious lattice. Upon applying current, the amorphous phase transformed into NiFeOOH, exhibiting a lattice spacing of 0.245 nm.^[^ ^27^ ^]^ Also, due to the reconstruction, numerous holey defects could be observed in F‐LDH/MX after OER which is beneficial for the ion diffusion and electron transport. Evidence for the reconstruction of F‐LDH/MX was also found in Raman spectra (Figure S12, Supporting Information). For LDH/MX, two characteristic peaks located at 449 and 539 cm^−1^ can be assigned to A_1g_ stretching modes of Ni─OH and Fe─O─Fe vibrations of NiFe‐LDH. No apparent peaks for MXene were found because it was fully covered by FeNi‐catalysts. After fluorination, the peaks of NiFe‐LDH disappeared and were replaced by two bulges at 425 and 497 cm^−1^, belonging to Ni─F and Ni─O in disordered Ni(OH)_2_ respectively. After oxygen evolution, three apparent peaks were observed. Among them, peaks centered at 477 and 554 cm^−1^ are attributed to the E_g_ bending and A_1g_ stretching vibrations of NiOOH, while the peak located at 675 cm^−1^ belongs to FeOOH. The results are in good agreement with TEM.

XPS was conducted to carefully study the surface chemical environment of F‐LDH/MX when immersed in an alkaline electrolyte and after the dynamic stability test. The high‐resolution spectra of Ni 2p showed that the peak of Ni 2p_3/2_ shifted from 656.76 to 656.08 eV after keeping in 1 m KOH without any potential (Figure 4f). The decrease in binding energy indicates that M‐F species are defluorinated even with no applied potential. Also, the value was close to that of LDH/MX (855.60 eV). It was reasonable to consider the formation of nickel hydroxide on the surface of F‐LDH/MX. After the stability test, the peak of Ni 2p_3/2_ shifted to a higher binding energy indicating the generation of metal oxyhydroxide in the presence of voltage. This is consistent with other reports on transition metal‐based catalysts, where metal oxyhydroxide is considered the real active site for catalyzing the OER.^[^ ^28^ ^]^ A similar change occurred in Fe 2p peaks, indicating the formation of iron hydroxide when immersed in 1 m KOH and the generation of oxyhydroxide during the OER process (Figure 4g). Negative shifts were also found on both Ni and Fe 2p_3/2_ peaks in F‐LDH after the OER because of the surface reconstruction. Compared with pristine LDH, Ni and Fe 2p_3/2_ peaks of F‐LDH after OER exhibited much higher binding energy proving the formation of M─OOH under high potentials (Figure S13, Supporting Information). Furthermore, positive shifts were observed for Fe and Ni peaks when compared with the XPS of F‐LDH/MX after OER, demonstrating the electron transfer between MXene and metal–oxyhydroxide (Figure S14, Supporting Information). In contrast, LDH/MX showed a marginal change in the binding energy of Ni/Fe peaks, and the binding energy of the peaks is lower than that of F‐LDH/MX after OER (Figure S15, Supporting Information). This clearly indicates that the metal species of F‐treated LDH on MXene are more readily activated to MOOH species than the M─OH species in LDH/MX, which is likely the main cause of the OER activity difference of the two catalysts. In the case of the Ti 2p spectra for F‐LDH/MX, the Ti─F species also transformed into Ti─O or F─Ti─O species (Figure S16a, Supporting Information). In the high‐resolution spectra of F 1s, though a strong peak was generated, which could be assigned to the C─F bond of the Nafion binder, the M─F bond becomes negligible further indicating the transformation from surface metal fluoride to metal oxyhydroxide in both F‐LDH and F‐LDH/MX (Figures S13d and S16b, Supporting Information).

It is widely considered that the surface lattice oxygen for catalysts is metastable, and catalysts with abundant oxygen vacancy are highly expected to facilitate the lattice oxygen redox chemistry.^[^ ^29^ ^]^ The XPS spectra for O 1s were further analyzed which can be characterized as M─O (≈529.49 eV), M‐OH (≈530.97 eV), oxygen vacancy V_O_ (≈531.85 eV), absorbed H_2_O (≈532.80 eV), respectively (Figure 4h). It was noted that the content of V_O_ increased significantly when the F element was introduced into LDH/MX from 22.68% to 43.43%. The same phenomenon was found in LDH and F‐LDH, changing from 18.19% to 44.08% (Figure S13, Supporting Information). After the OER, F element‐treated catalysts including F‐LDH and F‐LDH/MX exhibited a much higher content of V_O_ compared to LDH/MX (Figures S13 and S15, Supporting Information). This indicates that the in situ generated M─OOH in post‐OER F‐LDH/MX possesses more V_O_ than that on post‐OER LDH/MX. In addition, the content of oxygen vacancy in F‐LDH/MX enhanced after OER indicating that the lattice oxygen of in situ produced metal hydroxide after immersion in an alkaline solution was also involved in the oxygen evolution process. This increased V_O_ content in the F‐LDH/MX implies the OER is more likely to undergo the LOM than the AEM. This also explains why the OER overpotential for the F‐LDH/MX (251 mV) is lower than the minimum thermodynamic overpotential given by AEM (370 mV), and why the Tafel slope for the F‐LDH/MX (40.28 mV dec^−1^) is lower than that of the other prepared electrodes (>77.14 mV dec^−1^).^[^ ^30^ ^]^

To provide further evidence for the LOM on our catalysts, the pH‐dependent OER activity experiments were conducted (Figure 4i). It is reported that higher pH dependence of catalysts on the OER activity indicates the presence of nonconcerted proton–electron transfer steps or the LOM participation for OER activity.^[^ ^31^ ^]^ Hence, the activities for all samples were compared with varying pH at a potential of 1.55 V on the RHE scale. In addition, the slopes (r = (∂log i/∂pH)E) were calculated as a metric for the pH dependence. The F‐LDH/MX exhibited the highest value of ρ (1.026) as compared to LDH, (0.607), LDH/MX (0.616), and F‐LDH (0.775), indicating the strong participation of LOM during OER activity (Figure 4j). It is apparent that the fluorinated samples possess a higher value of ρ, which indicates that the in situ generated V_O_‐rich M─OOH tends to catalyze water oxidation via LOM. Hence, F‐LDH/MX exhibits substantially higher catalytic activity than LDH/MX.

**Figure 5:**
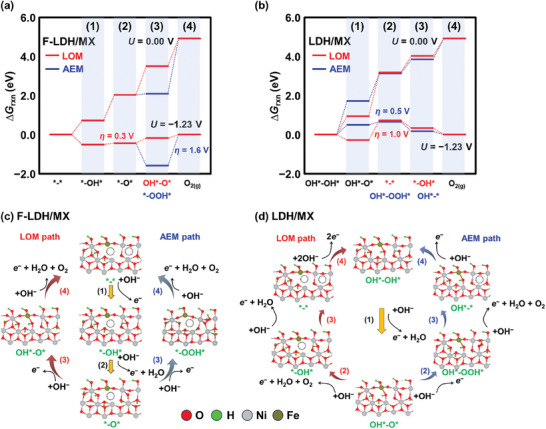
DFT calculated reaction energies of the OER at an applied potential of *U =* 0.00 V and *U =* −1.23 V for a) the activated F‐LDH/MX and b) the LDH/MX. The LOM and AEM mechanism pathways are calculated for activated c) F‐LDH/MX and d) LDH/MX. The overpotential(*η*) of each reaction pathway is denoted for the corresponding potential‐determining step. The color coding for the elements is as follows: O(red), H(green), Ni(gray), and Fe(olive).

To gain further insight into the reaction pathway of the OER on the catalysts, we conducted DFT simulations on the OER reaction pathways for both F‐LDH/MX and LDH/MX (**Figure** **5**; Figure S17, Supporting Information). We compare the overpotential(*η*) of each reaction pathway evaluated from the corresponding potential‐determining step (PDS) of the reaction pathway. Indeed, the abundance of V_O_ on the activated F‐LDH/MX surface promotes the LOM (*η* = 0.3 V, PDS: ^*^─O^*^ + OH^−^ *→* OH^*^─O^*^ + *e* ^−^) than the AEM (*η* = 1.6 V, PDS: ^*^─OOH^*^ + OH^−^ *→* ^*^ + ^*^ + O_2(g)_+H_2_O + *e* ^−^) on F‐LDH/MX. On the other hand, the AEM (*η* = 0.5 V, PDS: OH^*^─OH* + OH^−^ *→* OH^*^ + O^*^ + H_2_O + *e* ^−^) is significantly more feasible than the LOM (*η* = 1.0 V, PDS: OH^*^─OH^*^ + OH^−^ *→* OH^*^ + O^*^ + H_2_O + *e* ^−^) on the LDH/MX surface. These results are in excellent match with our pH‐dependent OER activity test where the F‐LDH/MX exhibited the highest pH‐dependency, hence the best LOM capability. These results clearly show the V_O_‐rich activated F‐LDH/MX significantly promotes the LOM, resulting in a substantially higher OER activity than the AEM‐dominant LDH/MX.

**Figure 6:**
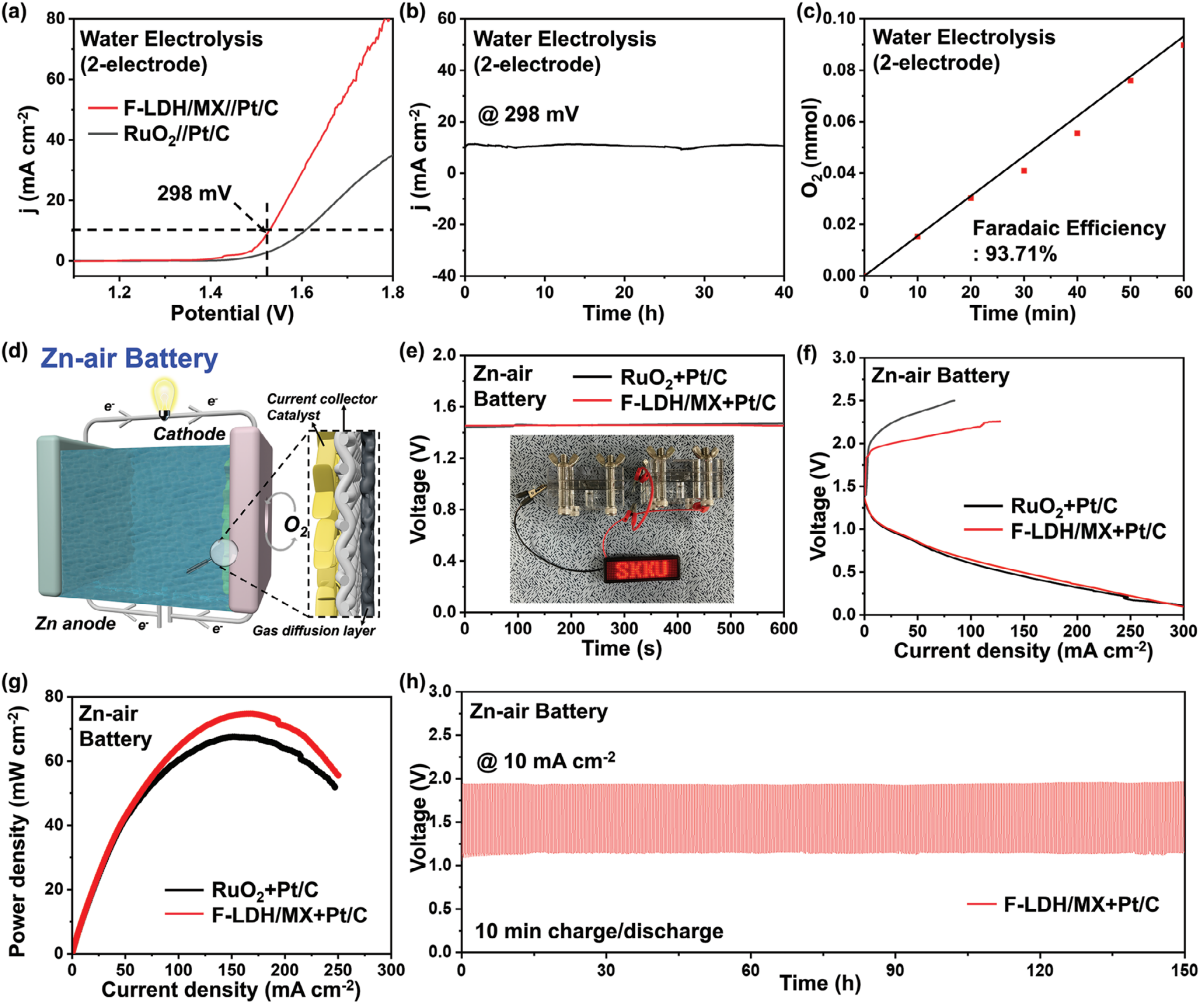
a) Overall water electrolysis polarization curves of the F‐LDH/MX//Pt/C and RuO_2_//Pt/C in 1 m KOH solution. b) Chronoamperometry curves of the F‐LDH/MX//Pt/C at the overpotential of 298 mV. c) Faradaic efficiency of F‐LDH/MX. d) Schematic of Zn‐air battery. e) OCP plots of aqueous Zn‐air batteries. (Inset: optical picture of a LED lighted by the Zn‐air battery of F‐LDH/MX+Pt/C). f) Polarization plots and g) power densities of aqueous Zn‐air batteries. h) Long‐term galvanostatic cycling tests at the current densities of 10 mA cm^−2^.

To simulate the actual application of the catalysts in water splitting, a two‐electrode system was constructed with the F‐LDH/MX‐modified electrode as the anode and a commercial Pt/C catalyst‐modified electrode as the cathode. A low potential of 1.528 V at 10 mA cm^−2^ was observed on this electrolyzer, indicating a small overpotential of 298 mV for overall water splitting in the alkaline medium, which was 82 mV lower than the standard RuO_2_//Pt/C electrolyzer (**Figure** **6**a). In addition, the stability of the system was investigated by long‐term CA in which no apparent decay was observed after 30 h (Figure 6b). The faradaic efficiency (F.E.) was also evaluated to rule out any false positives from side reactions and/or surface redox reactions (Figure 6c). The high F.E. value of 93.71% proves that most of the electron transfer at the anode was used to catalyze the oxygen evolution. In addition, F‐LDH/MX was employed in rechargeable a Zn‐air battery as well by paring it with Pt/C as the air electrode catalyst, with a Zn foil as the anode and 6.0 m KOH + 0.2 m Zn(Ac)_2_ as the electrolyte (Figure 6d). The F‐LDH/MX + Pt/C‐based Zn‐air battery operated well under a high open‐circuit voltage of 1.45 V, which lighted up the LED when two batteries were connected in series (Figure 6e). The polarization curves during the discharge and charge on the Zn‐air batteries are depicted in Figure 6f. The difference in the discharging curve between the different zinc‐air batteries was marginal because the discharge part is mainly dominated by the Pt/C catalyst. When it comes to the charging curves, F‐LDH/MX + Pt/C‐based battery delivered a current density of 25.7 mA cm^−2^ at 2V, showing a fivefold increase than that of RuO_2_ + Pt/C‐based battery (5.1 mA cm^−2^) indicating its better oxygen evolution performance. Moreover, F‐LDH/MX + Pt/C‐based battery exhibited a significantly higher power density of 75.43 mW cm^−2^ than that of the RuO_2_+Pt/C‐based battery (67.68 mW cm^−2^) (Figure 6g). The stability of the Zn‐air battery was also measured at the current density of 10 mA cm^−2^ (Figure 6h; Figure S18, Supporting Information). Initially, the RuO_2_ + Pt/C‐based battery delivered a voltage gap of 1.43 V with a round‐trip efficiency of 41.86%. After a 150 h test over 450 cycles, the performance decreased obviously with the voltage gap increasing to 1.86 V and the round‐trip efficiency decreasing to 27.06%. The stability changed when RuO_2_ was replaced with F‐LDH/MX. The F‐LDH/MX + Pt/C‐based battery initially delivered a small voltage gap of 0.83V with a round trip efficiency of 57.2%. After 150 h of measurement, no apparent performance degradation was observed for both the charge voltage and discharge voltage with a round trip efficiency of 58.6%, demonstrating excellent stability.

## 3 Conclusion

In summary, a 2D hybrid catalyst, consisting of MXene nanosheets coated with partially fluorinated LDH, was successfully synthesized via co‐precipitation and subsequent fluorination etching. The robust hierarchical structure of the electrocatalyst provides ample active surface area and a stable framework to prevent nanosheet agglomeration. The partially fluorinated LDH on MXene demonstrates a strong ability of self‐reconstruction to form real active sites, NiFeOOH, in alkaline water oxidation. Theoretical calculations and experiments show that the abundant oxygen vacancy in produced metal oxyhydroxide could activate the lattice oxygen and further enhance the participation of LOM, which leads to the remarkable electrochemical performance in OER. Notably, a low overpotential of 251 mV was required to derive the benchmark current density of 10 mA cm^−2^ with a Tafel slope of 40.28 mV dec^−1^, outperforming most MXene‐based non‐precious metal catalysts. In addition, the overall water‐splitting electrolyzer composed of F‐LDH/MX and Pt/C shows a low overpotential of 298 mV with robust stability for 30 h. The Zn‐air battery with F‐LDH/MX as the air electrode exhibits a high‐power density of 75.43 mW cm^−2^. This study suggests a promising and feasible approach to constructing oxygen‐vacancy‐rich catalysts for energy‐relevant electrochemical devices.

## Conflict of Interest

The authors declare no conflict of interest.

## Supporting information



Supporting Information

## Data Availability

Research data are not shared.
