# Supplementary material for: Fluorine‐Induced Lattice Oxygen Participation in 2D Layered Double Hydroxide/MXene Hybrids for Efficient Oxygen Evolution
Source: Adv Sci (Weinh). 2024 Nov 11;12(1):2410812. doi: 10.1002/advs.202410812 (PMC11714146; doi:10.1002/advs.202410812)
Supplement: Supplementary file 1 — Supporting Information [file ADVS-12-2410812-s001.docx]

Supporting Information

Fluorine-induced Lattice Oxygen Participation in 2D Layered Double Hydroxide/MXene Hybrids for Efficient Oxygen Evolution

*Chengang Pei, Min-Cheol Kim,* *Unbeom Baeck, Won Tae Hong, Jong Hun Kim, Hyungu Han, Jaekyum Kim, Sung Min Cho, Xu Yu, Jongwook Park*, Ho Seok Park*, and Jung Kyu Kim**

C. Pei, M.-C. Kim, U. Baeck, W. T. Hong, J. H. Kim, H. Han, J. Kim, Prof. S. M. Cho, Prof. H. S. Park, Prof. J. K. Kim

School of Chemical Engineering, Sungkyunkwan University (SKKU), 2066, Seobu-Ro, Jangan-gu, Suwon, 16419, Republic of Korea
E-mail: legkim@skku.edu (J. K. Kim) and phs0727@skku.edu (H. S. Park)

M.-C. Kim

Department of Chemistry, Sookmyung Women’s University, Seoul 04310, Republic of Korea

Prof. X. Yu

School of Chemistry and Chemical Engineering, Yangzhou University, Yangzhou, 225002, P. R. China

Prof. J. Park

Integrated Engineering, Department of Chemical Engineering, Kyung Hee University, Gyeonggi, 17104 South Korea

E-mail: jongpark@khu.ac.kr

Prof. H. S. Park

SKKU Institute of Energy Science and Technology (SIEST), Sungkyunkwan University (SKKU), Suwon, 16419, Republic of Korea

Prof. J. K. Kim

SKKU Advanced Institute of Nano Technology (SAINT), Sungkyunkwan University, 2066 Seobu-ro, Suwon 16419, Republic of Korea

**Experimental Section**

*Chemicals*: Nickel nitrate hexahydrate (Ni(NO_3_)_2_·6H_2_O), ferric nitrate nonahydrate (Fe(NO_3_)_3_·9H_2_O), urea, ammonium fluoride (NH_4_F), hydrofluoric acid (48%), and N-methyl pyrrolidone (NMP) were purchased from Sigma–Aldrich Chemical Co., Ltd. Dimethyl sulfoxide (DMSO) was obtained from Thermo Fisher Scientific Korea Ltd. Titanium aluminum carbide powder (98% purity) was supplied by SY Innovation, South Korea. All chemicals were used as received.

*Preparation of Ti_3_C_2_ MXene*: 20 mL of concentrated HF was added to 2.0 g of Ti_3_AlC_2_ MAX powder and stirred for 36 hours in a pre-heated oil bath at 50°C to etch the Al layer of the MAX phase. The etched MAX was then centrifuged several times to collect the multilayered MXene solution, followed by freeze-drying to turn it into powder. Next, 0.6 g of MXene powder was added to 20 ml of DMSO for intercalation and sonicated for four hours at room temperature. After sonication, the obtained delaminated MXene solution was stirred for 18 hours and washed several times with deionized water for the next steps.

*Preparation of FeNi-LDH/Ti_3_C_2_ MXene*: The Ni(NO_3_)_2_·6H_2_O (3 mmol), Fe(NO_3_)_3_·9H_2_O (1.5 mmol), and urea (12.0 g) were first dissolved in 20 mL of DI water. Another dark blue suspension was prepared by dispersing MXene colloidal solution into 30 mL of NMP. A mixture of the two solutions was then heated to 100°C under reflux for 5 h in Ar flow. The FeNi-LDH/Ti_3_C_2_ MXene (LDH/MX) were harvested by several centrifugation-rinsing cycles with deionized water followed by freeze-drying. FeNi-LDH can be obtained without adding the MXene colloidal solution.

*Preparation of* *partial fluorinated FeNi-LDH/MXene*: In a typical run, the as-obtained LDH/MX and NH_4_F were placed at two different positions on a porcelain boat. The mass ratio of LDH/MX to NH_4_F was 1:5. Subsequently, the porcelain boat was charged into a tube furnace with NH_4_F at the upstream side of the furnace, and the sample was heated at 150°C for 2 h under Ar flow. The final products were denoted as F-LDH/MX. The partial fluorinated FeNi-LDH (F-LDH) was prepared with FeNi-LDH as the precursor.

*Electrochemical Measurements*: The electrochemical measurements were performed on a Reference 600+ electrochemical workstation (GAMRY, USA) at room temperature (25°C). The OER performance was measured using a standard three-electrode cell. The glassy carbon electrode was used as the supporting working electrode, while a graphite rod and a Hg/HgO electrode were used as the counter and reference electrode, respectively. The preparation of the catalyst ink was as follows. Five milligrams of the as-prepared catalysts, 950μL of ethanol, and 50 μL of a Nafion solution were mixed and sonicated for 30 min to produce a homogeneous dispersion. Subsequently, 10 μL of the catalyst ink was loaded on GC and dried at room temperature.

The LSV curves were obtained by sweeping the potential from 1.0 to 1.7 V vs. RHE at room temperature with a sweep rate of 5 mV s^−1^. Tafel plots were recorded at a scan rate of 5 mV s^−1^ via LSV curves. Electrochemical impedance spectroscopy (EIS) was conducted in the frequency range from 1000 kHz to 0.01Hz. The durability test was carried out for 1000 cycles within the potential range of 1.1 to 1.7 V vs. RHE in 1 M KOH at a scan rate of 150 mV s^−1^, and a linear sweep was measured under a sweep rate of 5 mV s^−1^ after 1000 cycles. Chronoamperometry (CA) was carried out to estimate the stability of the catalyst at overpotential for 30 hours. The electrochemically active surface area (ECSA) was estimated from the electrochemical double-layer capacitance (C_dl_) by measuring the voltammograms at different scan rates in a potential window where no Faradaic processes occurred.

$$ECSA=\frac{C_{dl}}{C_{s}} (1)$$

The general specific capacity (C_s_) of 0.040 mF cm^−2^ was used to calculate the ECSA.

The Turnover frequency (TOF) values were calculated using the following equation:

$$TOF=\frac{j\times A}{4\times F\times m} (2)$$

where *j* is the current density, *A* is the surface area of the electrode, *F* is the Faraday constant (96485 C mol^−1^), and *m* is the number of moles of active materials.

In alkaline electrolytes, pH-dependent OER kinetics follow below equation:

$$i=\theta C_{OH}e^{-\Delta G/RT} (3)$$

where i, θ, C_OH_, ΔG, R, and T are the OER current, the surface coverage of the adsorbed *OH and *OOH intermediates, the concentration of OH^−^, the free energy, the gas constant, and the temperature during experiments, respectively. By varying the pH, the OER activity can be tuned by changing the free energy of adsorbed intermediates, the concentration of OH^−^, or the surface coverage of the adsorbed *OH and *OOH intermediates.

The water splitting measurements were conducted as follows. The overall water splitting was carried out in a two-electrode system. The F-LDH/MX and commercial RuO_2_ electrocatalyst were used as the anode electrode, while the Pt/C was used as the cathode electrode. LSV was performed at a scan rate of 5 mV s^−1^ over a potential range of 1.0−2.0 V (vs. RHE). Chronoamperometry (CA) was carried out to estimate the stability of the catalyst at an overpotential for 30 hours.

The Faradaic yield was calculated from the ratio of the recorded gas volume to the theoretical gas volume during the charge passed through the electrode. The formula is listed below:

$$Faradaic yield=\frac{V_{\mathrm{experimental}}}{V_{\mathrm{theoretical}}}=\frac{V_{\mathrm{experimental}}}{\frac{1}{4}{\times\frac{Q}{F}\times V}_{m}} (4)$$

where *Q* is the charge passed through the electrode, *F* is the Faraday constant (96485 C mol^−1^), the number 4 means 4 moles of electrons per mole O_2_, and *V_m_* is the molar volume of gas (24.6 L mol^−1^, 298 K, 101 K Pa).

*Characterization*: The structures and morphologies of the samples were investigated with field-emission SEM (FESEM, JSM-7600F) and high-resolution transmission electron microscope (HR-TEM, JEM ARM 200F) with an accelerating voltage of 200 kV. Energy dispersive X-ray (EDX) elemental maps were obtained using a scanning TEM model. Powder X-ray diffraction (XRD) was performed on a Bruker D8 Advance X-ray diffractometer using Cu Kα radiation. The elementary constituents of the samples were determined using X-ray photoelectron spectroscopy (XPS, ESCALAB250), and the C 1s photoelectron peak at 284.8 eV to calibrate the binding energies. The surface wettability of the catalyst-modified electrodes was examined using a DSA25E Drop Shape Analyzer.

*Density Functional Theory Calculations*: All DFT calculations were performed using the Vienna *Ab Initio* Simulation Package (VASP),^[1]^ with the revised Perdew–Burke–Ernzerhof (RPBE)^[2]^ functional as the exchange and correlation functional with the D3 dispersion correction.^[3]^ The projector-augmented-wave method was adopted to describe the potential from the ionic core and an energy cutoff of 400 eV was used. Monkhorst-Pack *k*-point sampling with *k*-point spacing < 0.03 Å^−1^ was used for all systems. Spin polarization and dipole corrections were also included.

We modeled the F-LDH/MX catalyst as a NiFeOOH slab vertically attached to a partially fluorinated MXene monolayer slab (Figure S17a), while LDH/MX is modeled as the NiFeOOH vertically attached to the pristine MXene monolayer slab (Figure S17b). The F and Fe dopant position was selected as the position with the lowest energy among all possible configurations. All atoms in the F-LDH or LDH slab were allowed to relax until the forces on the individual relaxed atoms were less than 0.05 eV Å^-1^, whereas the MXene layer was fixed during the relaxation. For the catalytic active site, the basal Ni site pair adjacent to Fe is chosen. We investigated the basal plane sites as they are more abundant than the edge sites. The basal Ni site pair adjacent to Fe had the longest Ni-O bond length (>2.3 Å) among the metal sites (with Ni-O bonds of ~2.2 Å and ~1.92 Å) which indicates highly catalytically active sites. In addition, Ni sites neighboring Fe dopants have been frequently reported as OER active sites in NiFe-LDH catalysts.^[4]^

To calculate the Gibbs free energy change for each step, the potential under standard reaction conditions (298 K and 1 atm) was set as the reference potential, in which the chemical potential of the (H^+^ + *e^−^*) pair was related to half of the chemical potential of a hydrogen gas molecule (H_2_) at pH 0.^[5]^ For each step, Δ*G* = Δ*H + T*Δ*S +*Δ*E*_ZPE_ *+* Δ*G*_U_. Here, the enthalpy *H* = *E* + *E*_ZPE_ + $\int_{0}^{T} C_{V}dT$, where *E* is the electronic energy, *E*_ZPE_ is the zero-point correction energy, and the last term is the integration of the heat capacity. *T* is the absolute temperature and *S* is the entropy. *E*_ZPE_, $\int_{0}^{T} C_{V}dT$, and *S* can all be derived from vibrational frequencies of adsorbed species, which are evaluated from finite difference calculations. We adopted free energy corrections of the reference gas molecules (H_2_ and H_2_O) from the experimental data. Due to the intrinsic errors in the DFT for describing O_2(g)_ energy, the energy of O_2(g)_ is obtained from the formation energy of O_2_ with respect to water.^[6]^ Δ*G*_U_ represents the effect of the applied bias and is equal to –*neU*, where n is the number of transferred electrons in each step.


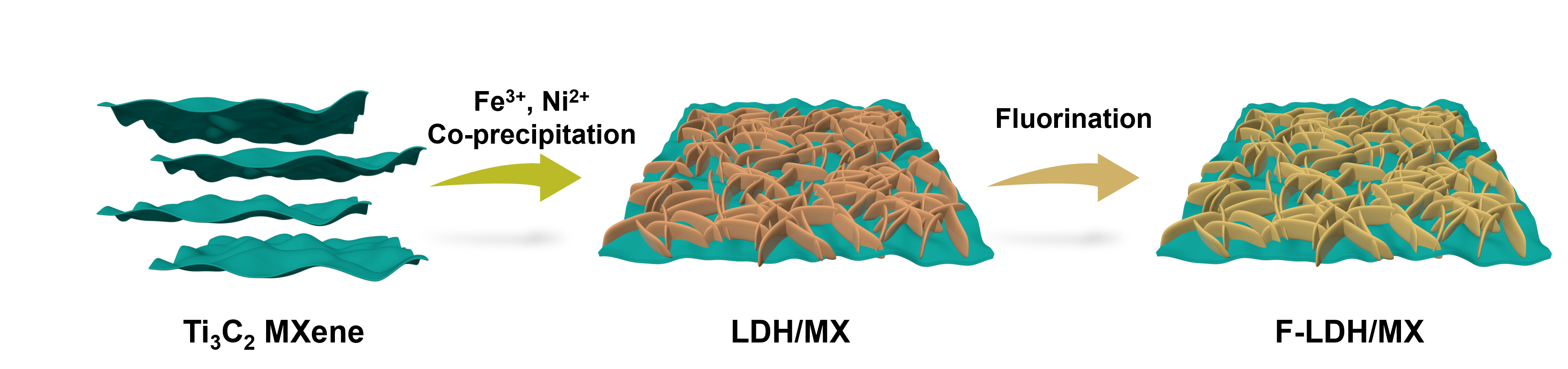


**Figure S1.** Schematic of preparation for F-LDH/MX.


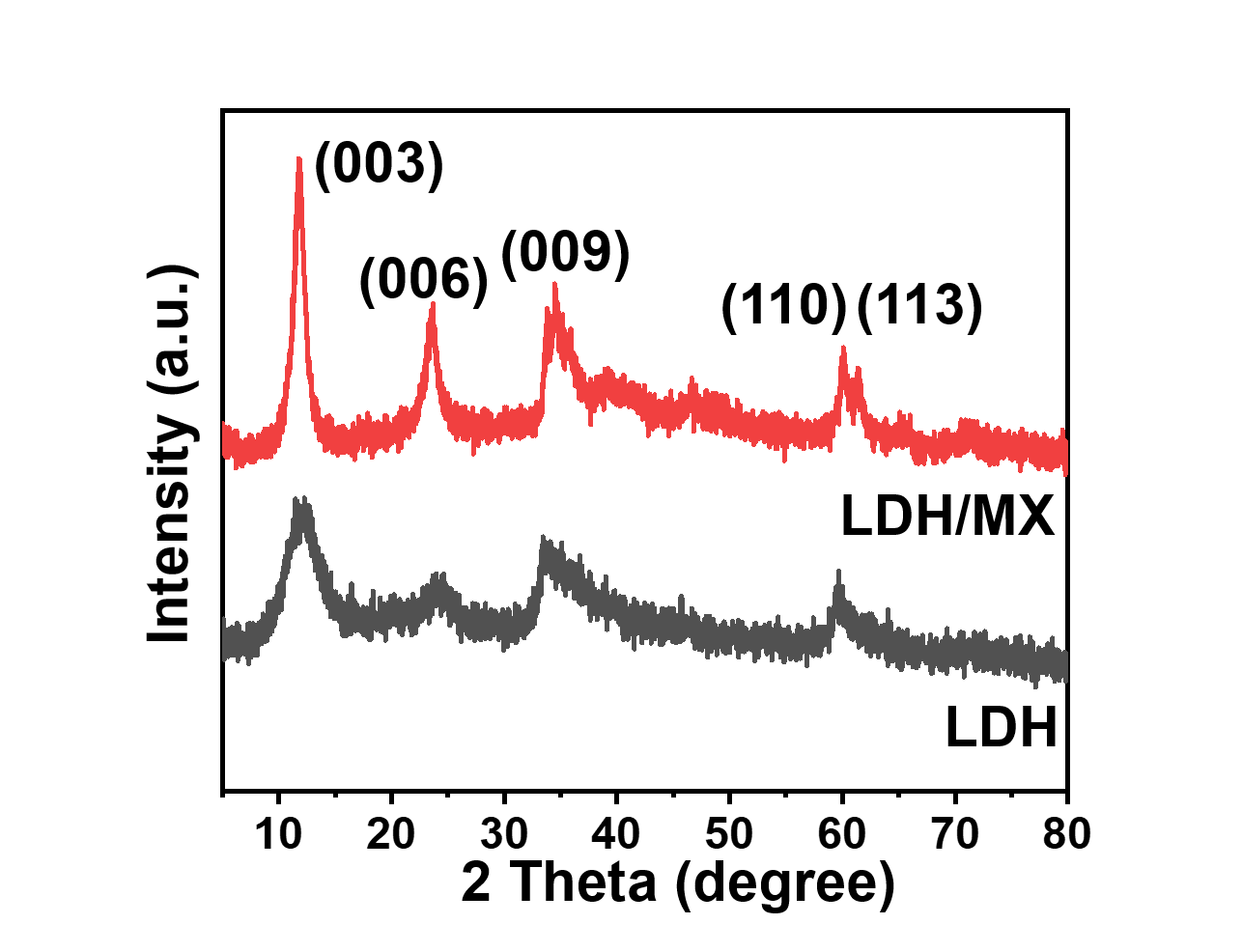


**Figure S2.** XRD patterns of the LDH and LDH/MX samples.

**
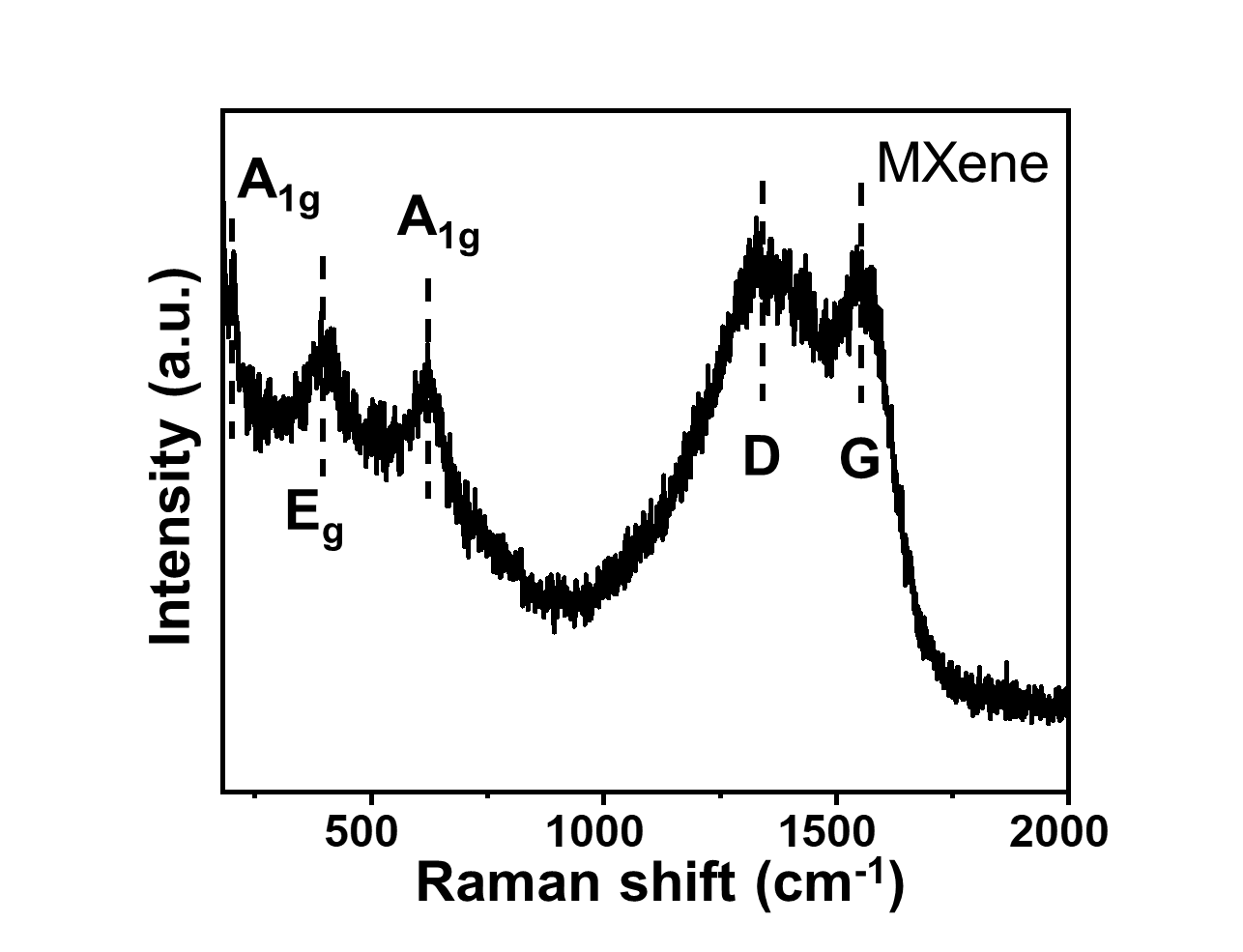
**

**Figure S3.** Raman spectrum of Ti_3_C_2_ MXene.


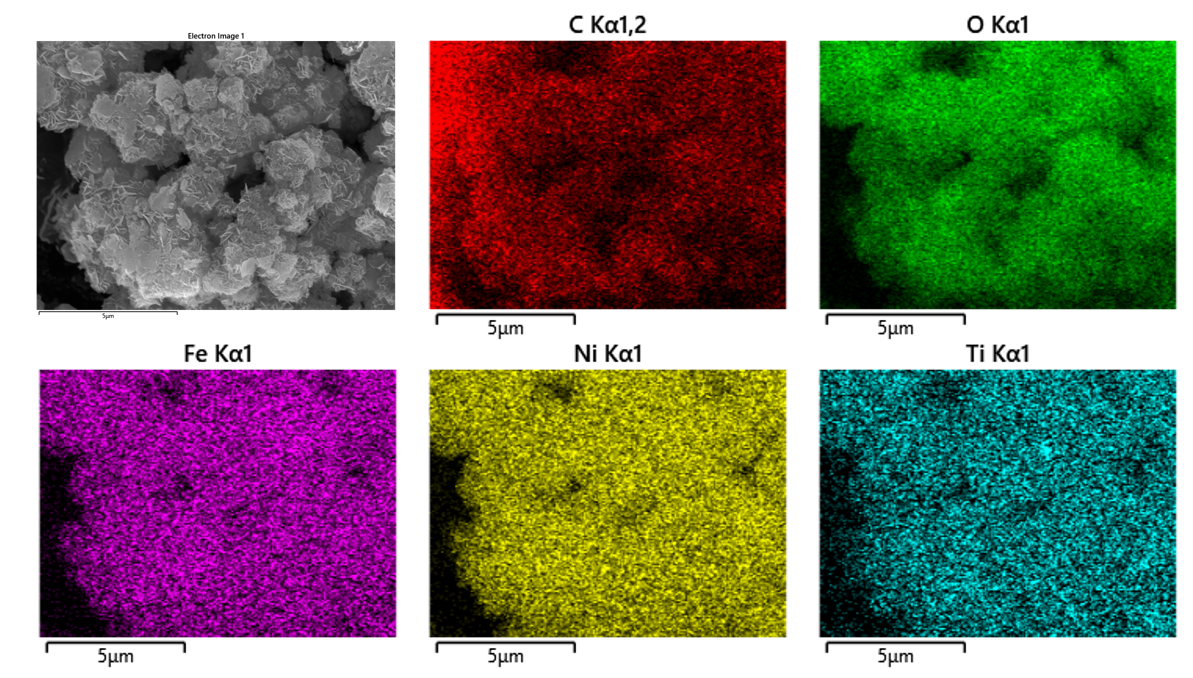


**Figure S4.** EDS mapping of LDH/MX.


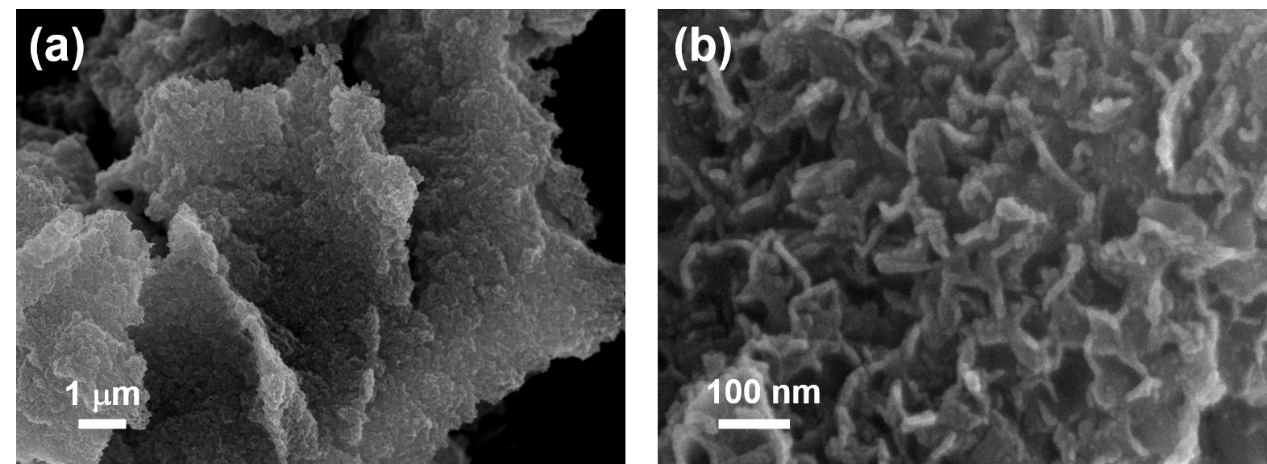


**Figure S5.** SEM images of LDH.


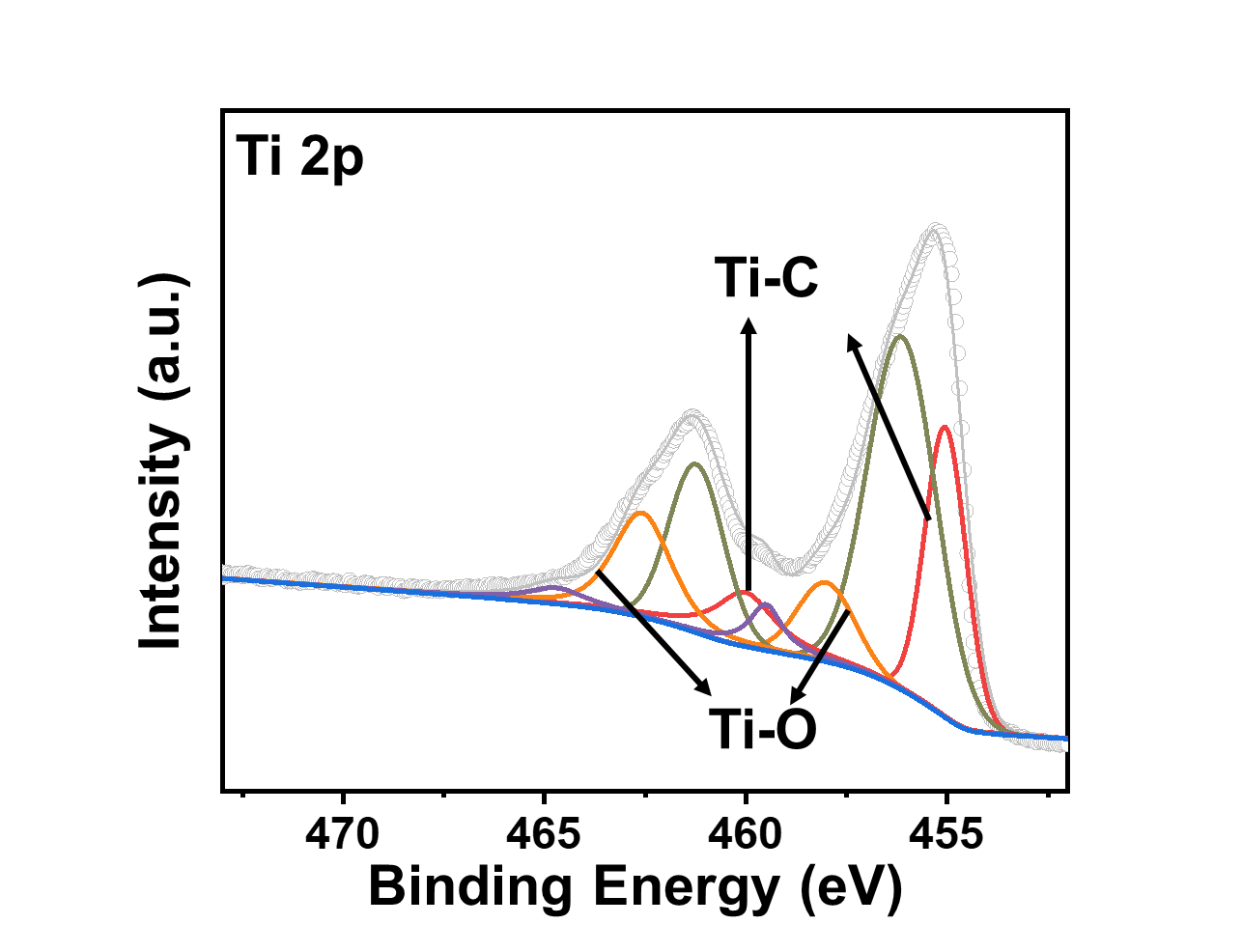


**Figure S6.** High-resolution fitted XPS spectra of Ti 2p for 2D MXene.


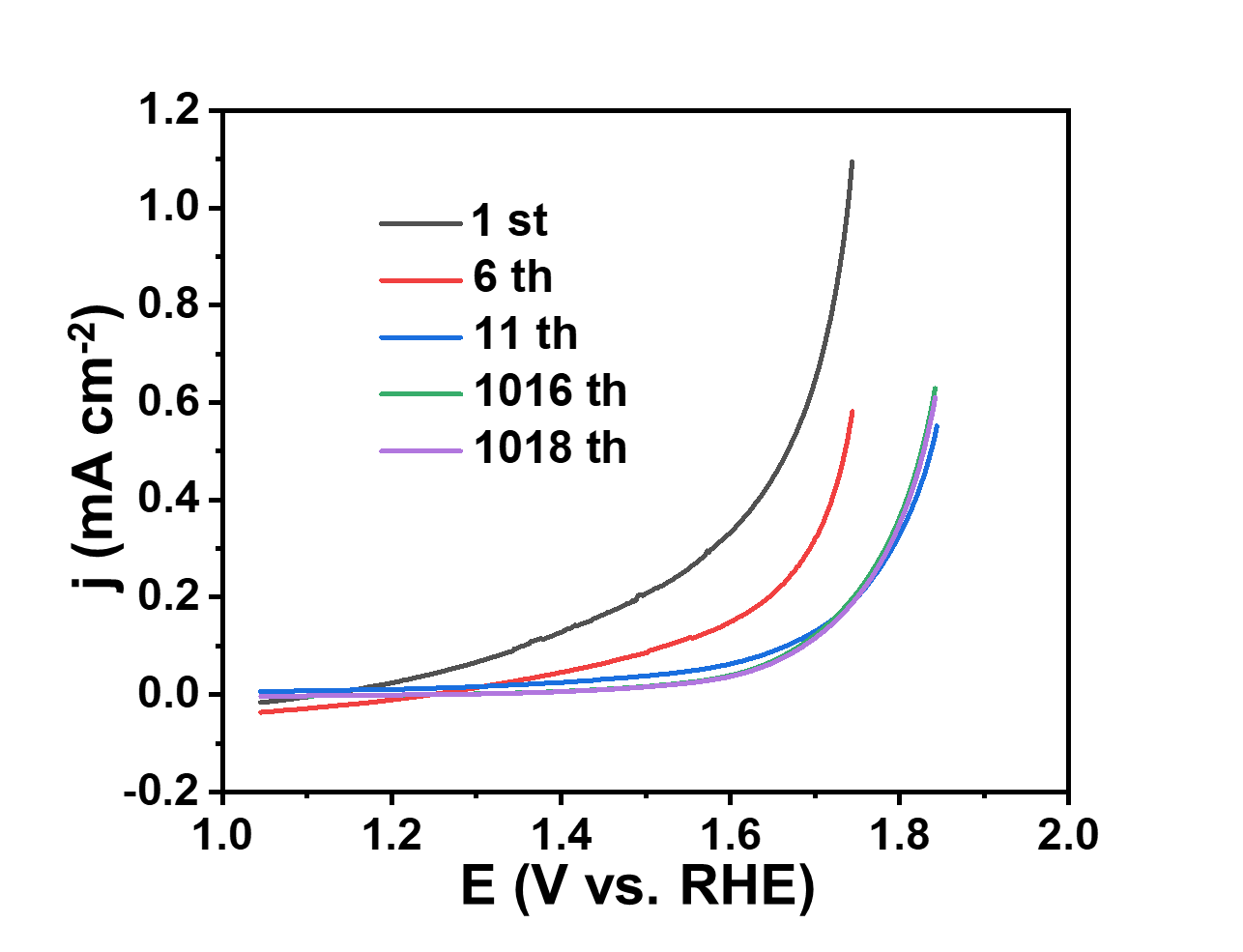


**Figure S7.** Polarization curves of MXene at different cycles in 1 M KOH.


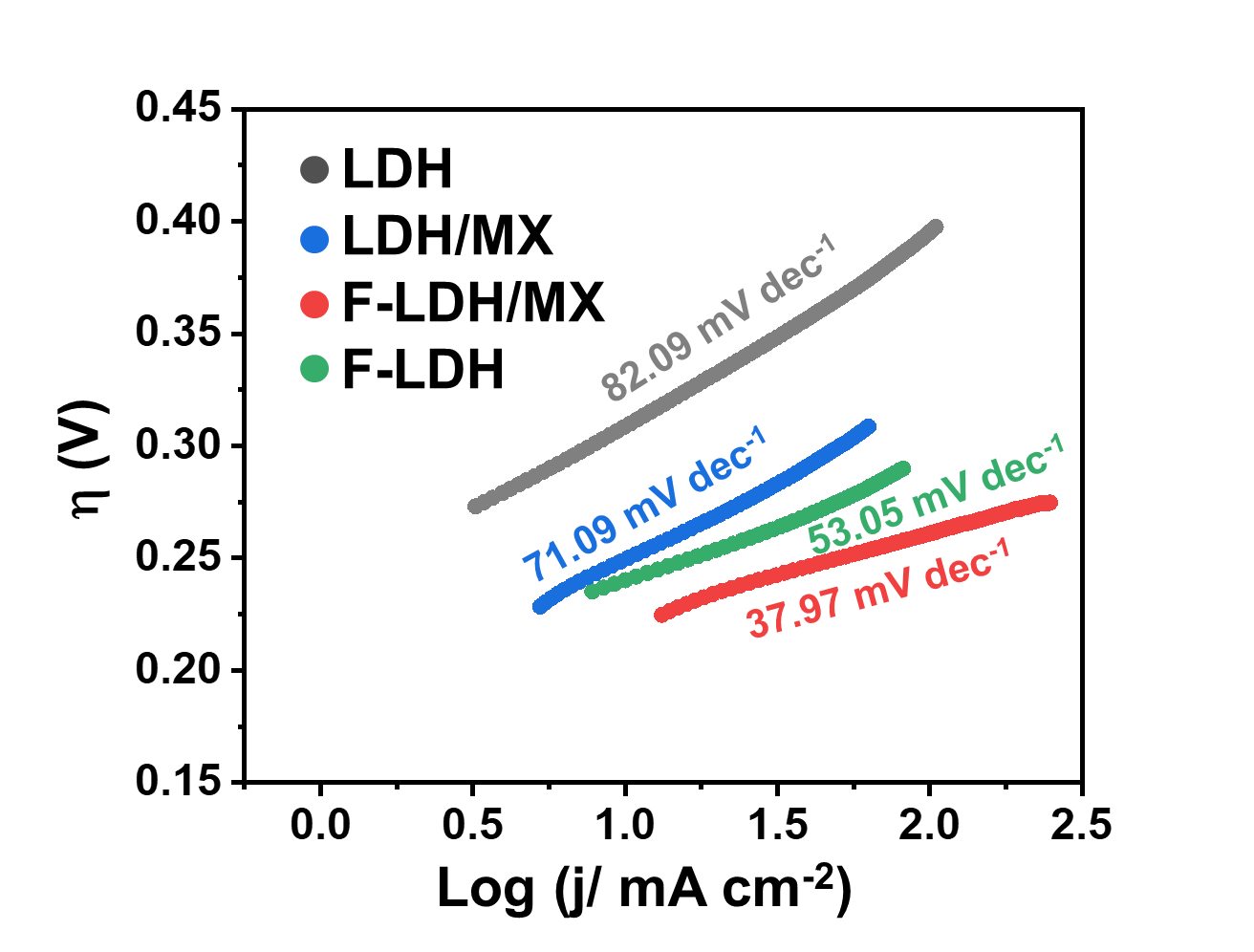


**Figure S8**. Tafel slope of LDH, LDH/MX, F-LDH/MX, and samples loaded on NF in 1 M KOH.


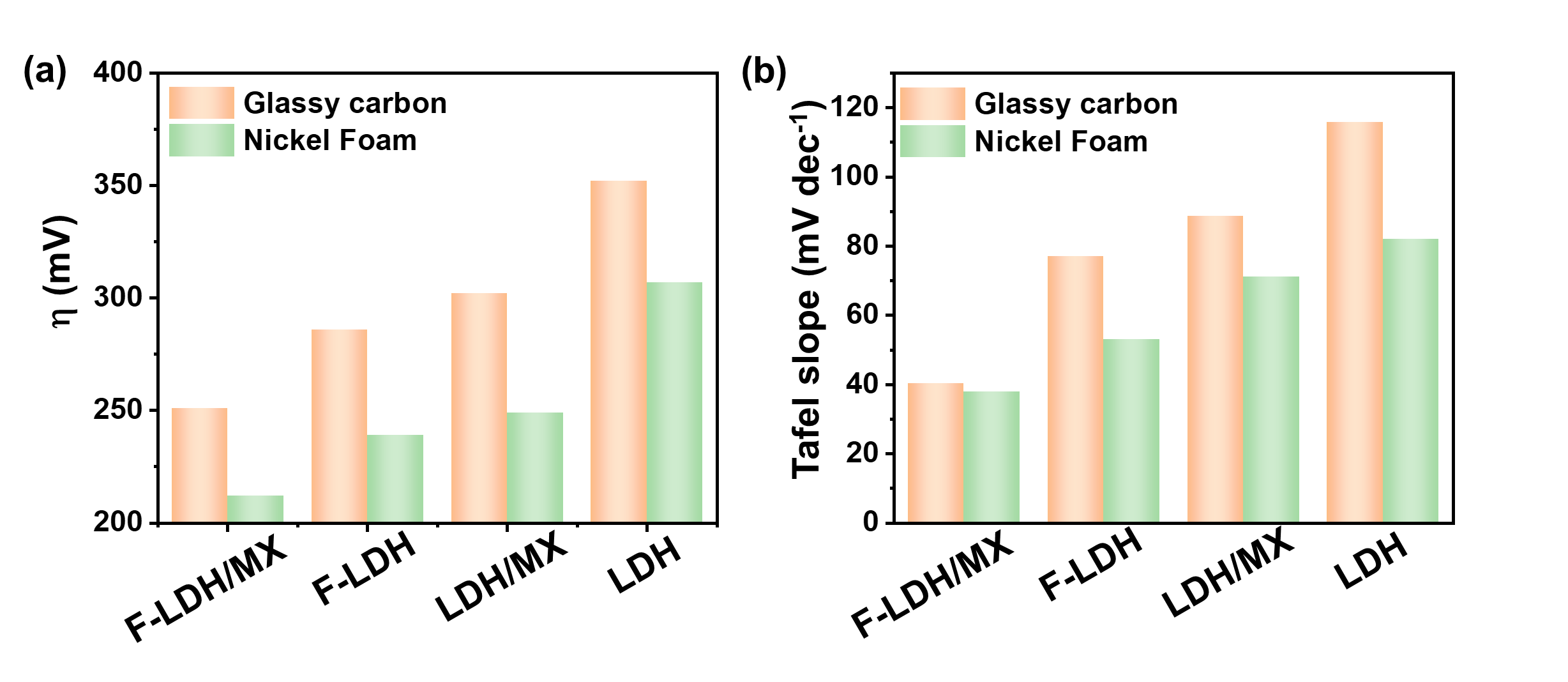


**Figure S9.** The comparison of (a) overpotential and (b) Tafel slope loaded on GC and NF in 1 M KOH.


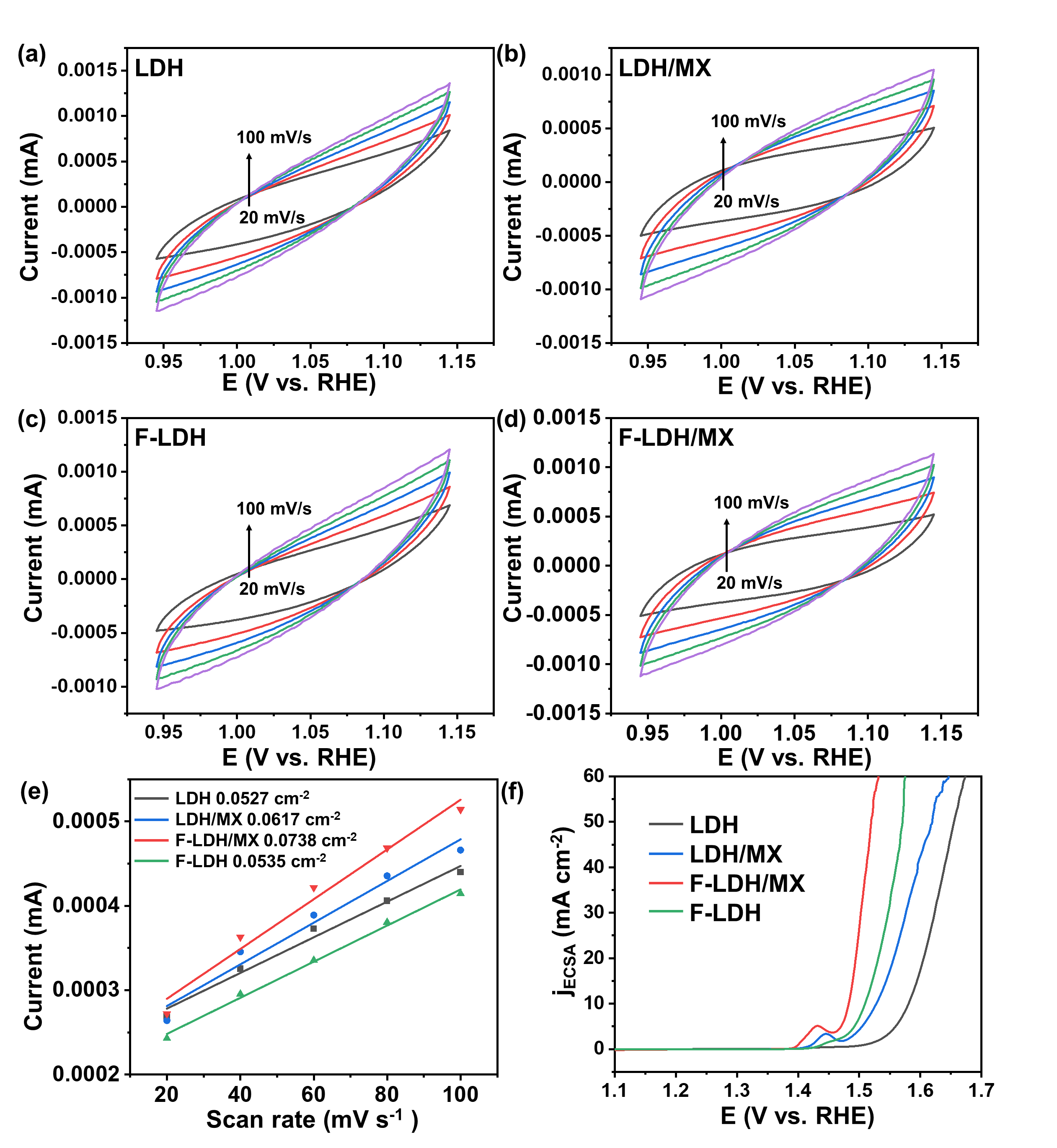


**Figure S10.** Scan-rate dependent CVs of (a) LDH, (b) LDH/MX (c) F-LDH and (d) F-LDH/MX, respectively at the potential of 0.945 V-1.145 V in 1 M KOH. (e) Linear plot of capacitive current vs. scan rate for these samples. (f) The specific activity of samples.

**
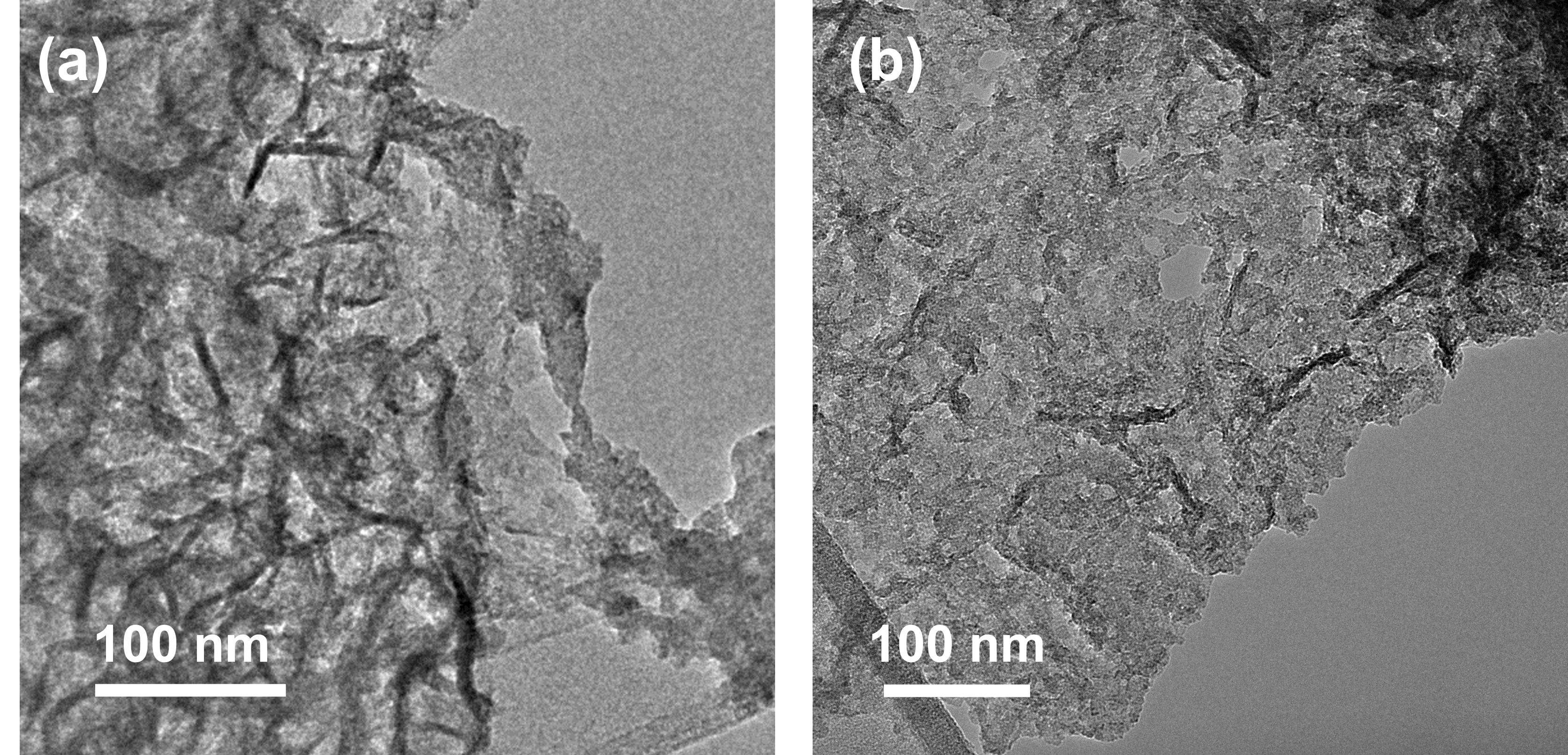
**

**Figure S11.** The TEM images of (a) F-LDH/MX in KOH and (b) F-LDH/MX after OER.


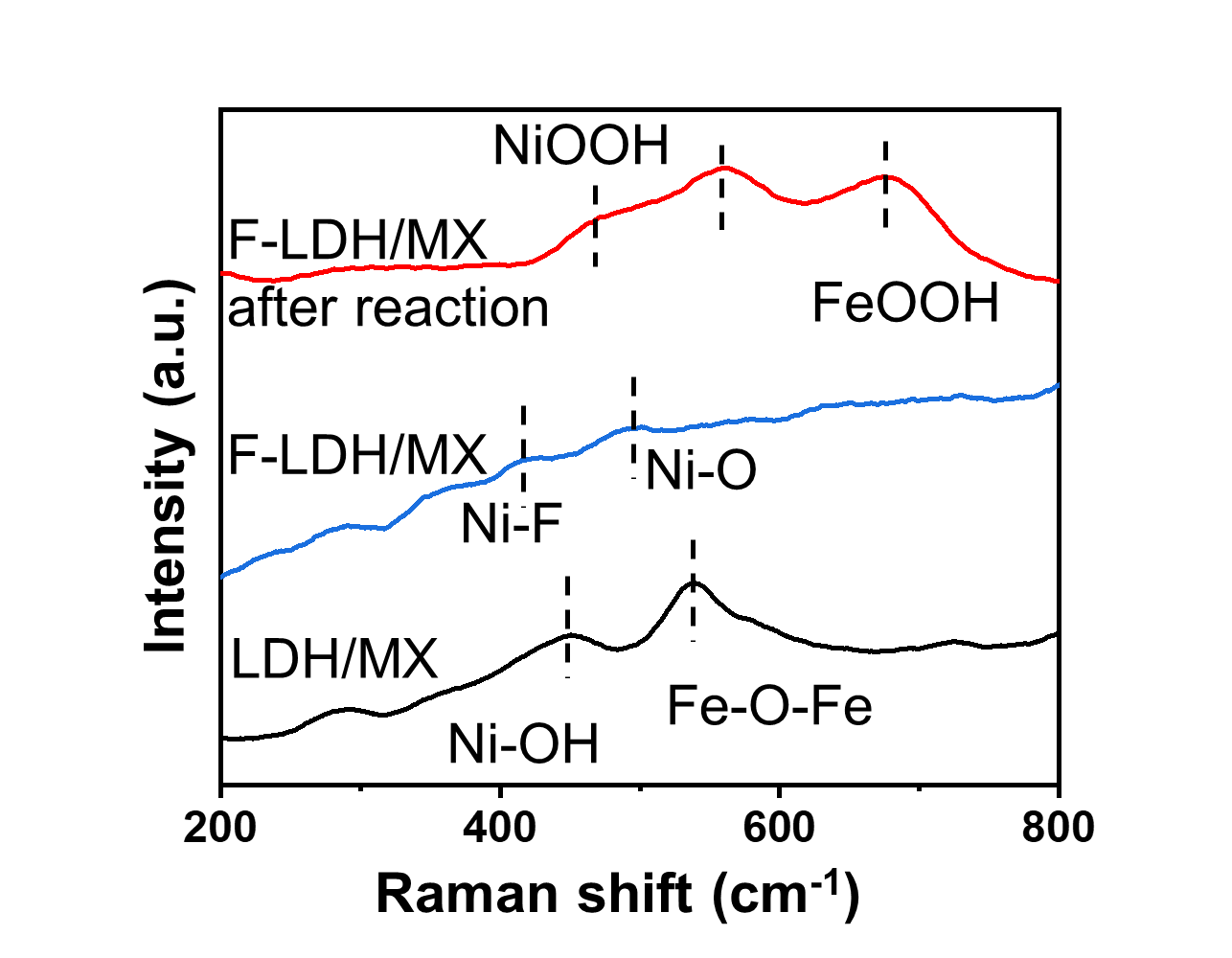


**Figure S12.** Raman spectrum of samples.


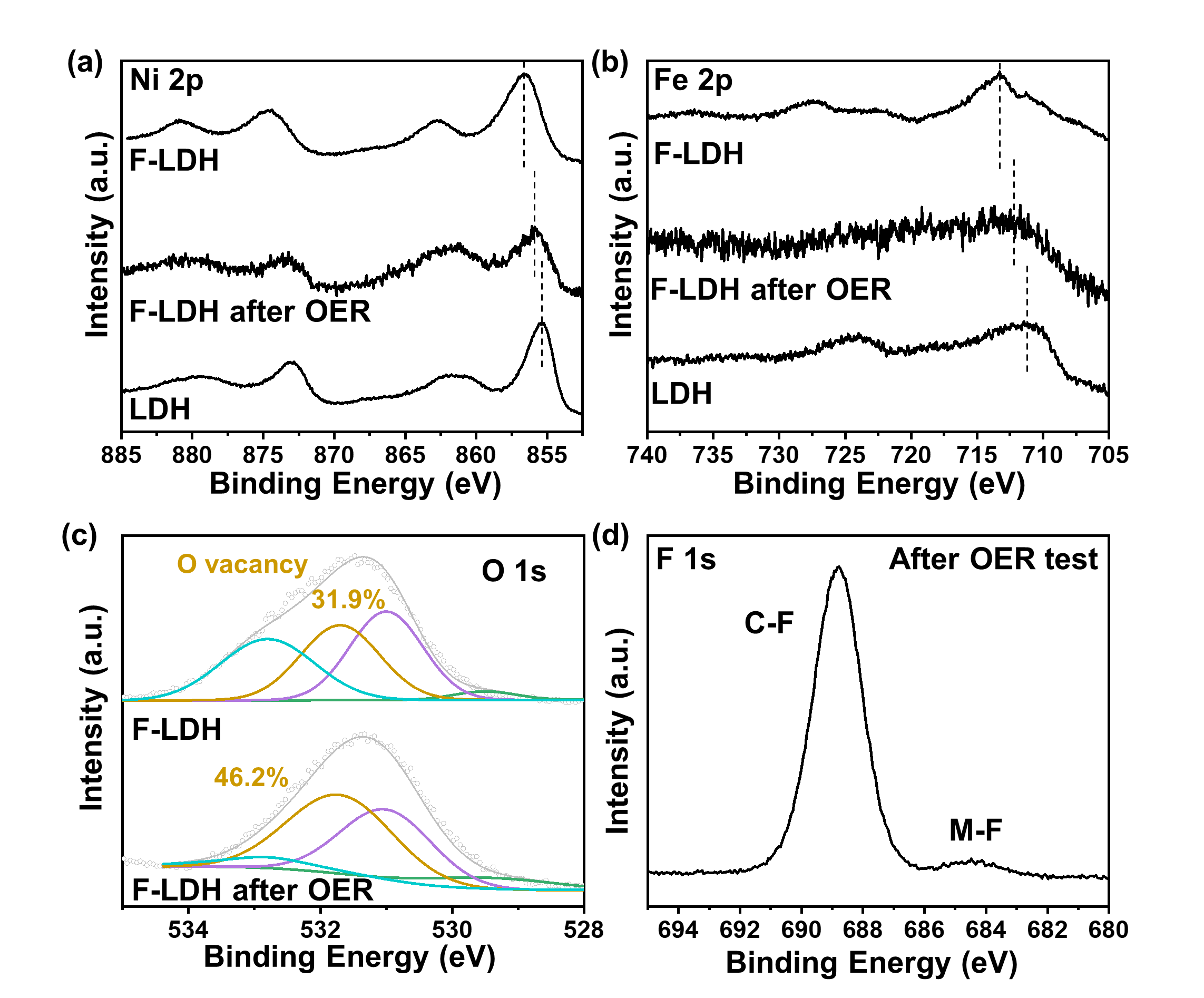


**Figure S13.** The high-resolution spectra of (a) Ni 2p, (b) Fe 2p, (c) O 1s and (d) F 1s for F-LDH and F-LDH after OER.


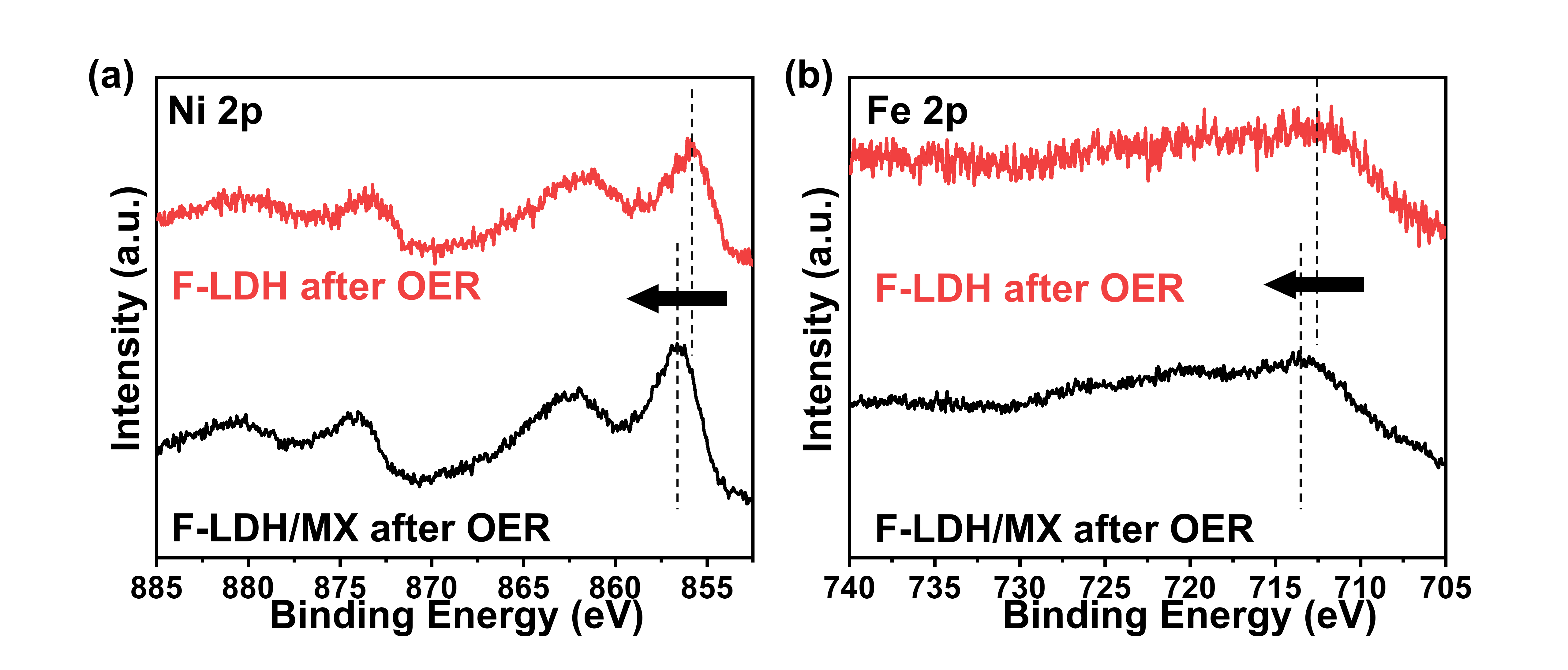


**Figure S14.** The comparison of high-resolution Ni and Fe XPS for F-LDH and F-LDH/MX after OER.


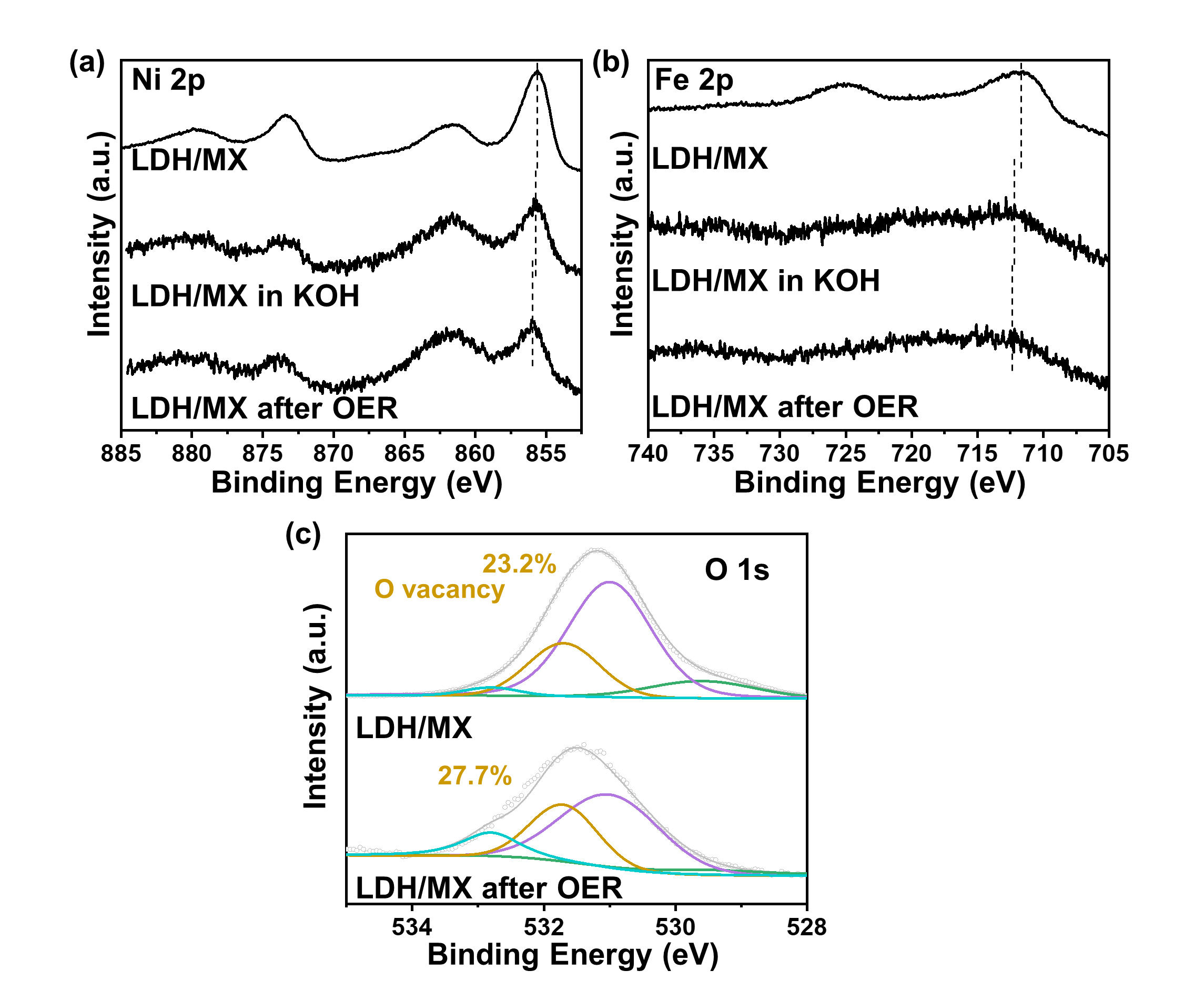


**Figure S15.** The high-resolution spectra of (a) Ni 2p, (b) Fe 2p and (c) O 1s for LDH/MX and LDH/MX after OER.


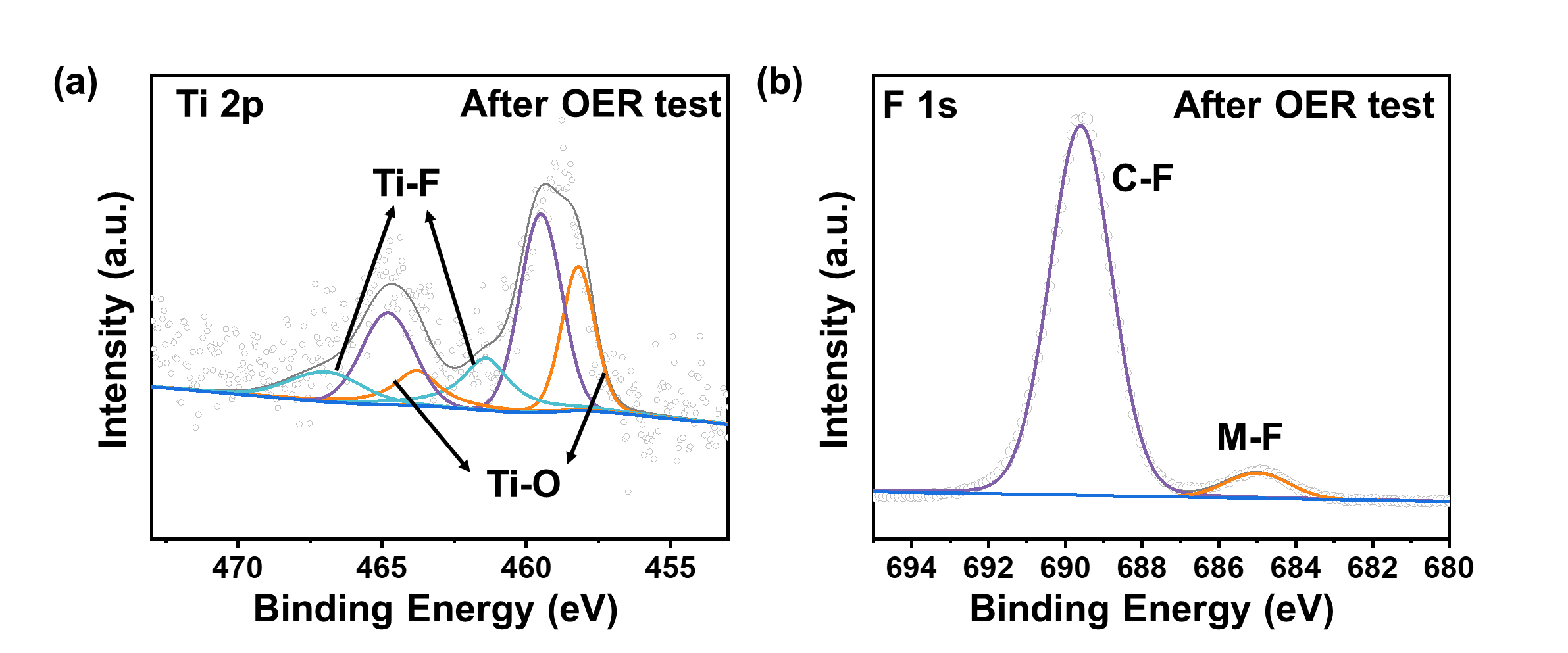


**Figure S16.** The high-resolution spectra of (a) Ti 2p and (b) F 1s for F-LDH/MX after OER.


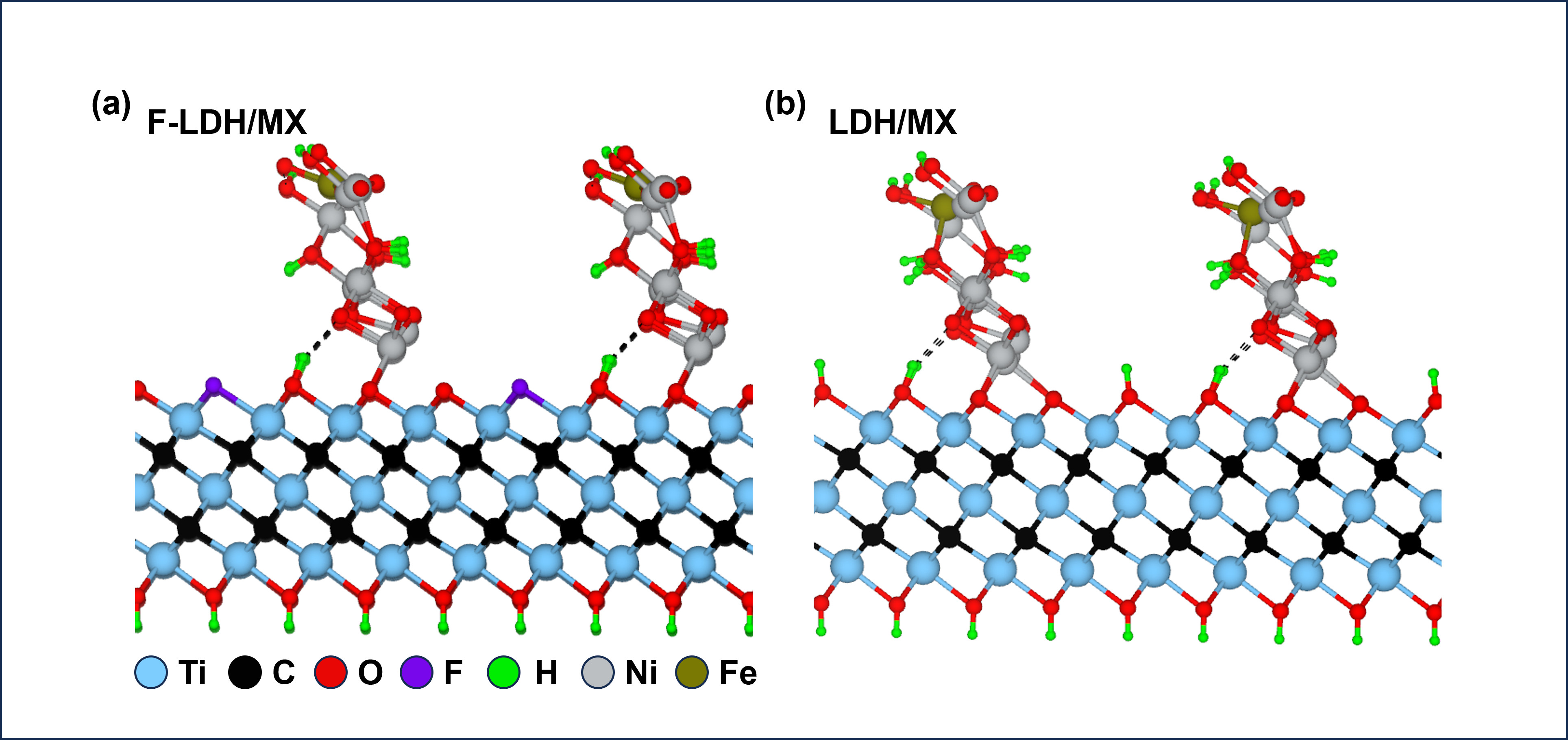


**Figure S17.** Molecular structure of (a) the F-LDH/MX and (b) LDH/MX after OER used in the DFT simulations.

**
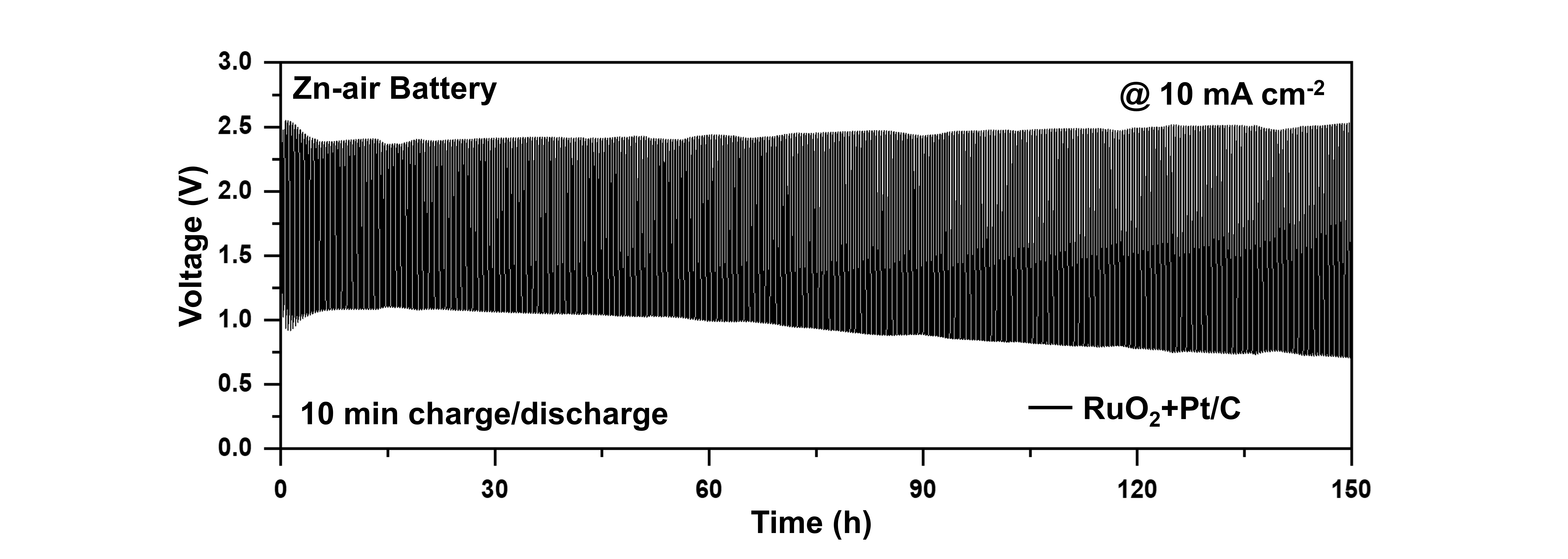
**

**Figure S18.** Long-term galvanostatic cycling test of Zn-air battery based on RuO_2_+Pt/C at the current densities of 10 mA cm^-2^.

**Table S1**. The atomic content of each element for LDH/MX and F-LDH/MX from XPS.

| Element | LDH/MX | F-LDH/MX |
| --- | --- | --- |
| C | 9.22 % | 7.96 % |
| O | 52.4 % | 13.93 % |
| N | 3.53 % | 12.12 % |
| Fe | 10.43 % | 8.63 % |
| Ni | 21.35 % | 18.65 % |
| F | 2.05 % | 37.89 % |
| Ti | 1.03 % | 0.82 % |

**Table S2**. EIS fitting parameters from equivalent circuits for LDH, LDH/MX, F-LDH, and F-LDH/MX.

|  | R_s_ | R_ct_ | R_1_ |
| --- | --- | --- | --- |
| LDH | 11.70 | 1067 | 102.8 |
| LDH/MX | 12.29 | 112.5 | 47.21 |
| F-LDH/MX | 10.86 | 24.97 | 6.62 |
| F-LDH | 9.944 | 48.77 | 46.44 |

**Table S3**. The comparison of OER performances with other MXene-based catalysts in 1 M KOH.

| Sample | Overpotential at  10 mA cm^-2^ (mV) | Tafel slope  (mV dec^-1^) | Substrate | Electrolyte | Ref. |
| --- | --- | --- | --- | --- | --- |
| F-LDH/MX | 251 | 40.28 | GC | 1 M KOH | This |
|  | 212 | 37.97 | NF | 1 M KOH | work |
| CoS_2_@MXene | 270 | 92 | NF | 0.1 M KOH | ^[7]^ |
| NiFeP/MXene | 286 | 35 | GC | 1 M KOH | ^[8]^ |
| FeNi-LDH/Ti_3_C_2_ | 298 | 43 | GC | 1 M KOH | ^[9]^ |
|  | ~230 | 48 | NF | 1 M KOH | ^[9]^ |
| Ti_3_C_2_T_x_-CoBDC | 410 | 48.2 | GC | 0.1 M KOH | ^[10]^ |
| CoP/Ti_3_C_2_ | 280 | 95.4 | GC | 1 M KOH | ^[11]^ |
| Co-CoO/Ti_3_C_2_ | 271 | 47 | NF | 1 M KOH | ^[12]^ |
| CoP@3D Ti_3_C_2_ | 320 | 59 | GC | 1 M KOH | ^[13]^ |
| Ti_3_C_2_T_x_/TiO_2_/NiFeCo-LDH | 320 | 98.4 | GC | 0.1 M KOH | ^[14]^ |
| Co_3_O_4_ QDs/MXene | 340 | 63.97 | GC | 1 M KOH | ^[15]^ |
| CoFe-LDH/MXene | 319 | 50 | GC | 1 M KOH | ^[16]^ |
| CoP/MXene | 230 | 50 | CFP | 1 M KOH | ^[17]^ |
| NiFeCoP/MXene | 240 | 55 | NF | 1 M KOH | ^[18]^ |
| M_3_OOH@V_4_C_3_Tx | 275.2 | 51.4 | GC | 1 M KOH | ^[19]^ |
| FeCo-LDH/MXene | 268 | 108 | GC | 1 M KOH | ^[20]^ |
| NiMn-LDH/Ti_3_C_2_ | 294 | 83.7 | GC | 1 M KOH | ^[21]^ |
| NiCoS/Ti_3_C_2_T_x_ | 365 | 58.2 | GC | 1 M KOH | ^[22]^ |

Note: GC means Glassy carbon; NF means Nickel foam; CFP represents Carbon Fiber.

**Table S4**. The comparison of OER performances with state-of-the-art NiFe-based OER electrocatalysts in 1 M KOH.

| Sample | Overpotential at  10 mA cm^-2^ (mV) | Tafel slope  (mV dec^-1^) | Substrate | Electrolyte | Ref. |
| --- | --- | --- | --- | --- | --- |
| F-LDH/MX | 251 | 40.28 | GC | 1 M KOH | This |
|  | 212 | 37.97 | NF | 1 M KOH | work |
| FeNi-LDH/Ti_3_C_2_ | ~230 | 48 | NF | 1 M KOH | ^[9]^ |
| a-LNFBPO | 215 | / | NF | 1 M KOH | ^[23]^ |
| TA-Ni_3_Fe | 290 | 28 | CP | 1 M KOH | ^[24]^ |
| NiFe_0.2_-O_x_H_y_ | 263 | 35 | GC | 1 M KOH | ^[25]^ |
| NiFe-DASC | 310 | 45 | GC | 1 M KOH | ^[26]^ |
| Ni_0.75_Fe_0.25_Se_2_ | 267 | 67 | GC | 1 M KOH | ^[27]^ |
| AB@NiFeS_x_(3:2) | 232.2 | 35.43 | NF | 1 M KOH | ^[28]^ |
| NiFeO_x_(OH)_y_@MoS_2_/rGO | 245 | 34 | CP | 1 M KOH | ^[29]^ |
| NiFe-LDH/MXene/NF | 229 | 44 | NF | 1 M KOH | ^[30]^ |
| Ni(Fe)OOH-Fe_2_O_3_ | ~230 | 41 | NF | 1 M KOH | ^[31]^ |
| CCS-NiFeP-10 | 201 | 41.2 | CC | 1 M KOH | ^[32]^ |
| (Ni_0.77_Fe_0.23_)Se | 228 | 69 | CC | 1 M KOH | ^[33]^ |
| E-NiFeOOH-40 min | 215 | 30.7 | NF | 1 M KOH | ^[34]^ |
| P-doped Ni–Fe–S | 264 | 48 | GC | 1 M KOH | ^[35]^ |
| NiFe/MoS_2_ | 260 | 48 | GC | 1 M KOH | ^[36]^ |
| Engraved NiFe-LDH(30s) | 250 | 69 | NF | 1 M KOH | ^[37]^ |
| NiFeSe@NiSe\|O@CC | 270 | 63.2 | CC | 1 M KOH | ^[38]^ |
| 10.1-NiFe | 307 | 112 | Silicon | 1 M KOH | ^[39]^ |
| Ni_0.67_Fe_0.33_/C | 230 | 35.1 | CFP | 1 M KOH | ^[40]^ |
| FeNiF/NCF | 260 | 67 | GC | 1 M KOH | ^[41]^ |

Note: GC means Glassy carbon; NF means Nickel foam; CFP represents Carbon Fiber; CP means carbon paper.

Reference

[1] G. Kresse, J. Furthmüller, *Comput. Mater. Sci.* **1996**, *6*, 15.

[2] B. Hammer, L. B. Hansen, J. K. Nørskov, *Phys. Rev. B* **1999**, *59*, 7413.

[3] S. Grimme, J. Antony, S. Ehrlich, H. Krieg, *J. Chem. Phys.* **2010**, *132*.

[4] a) J. Jiang, F. Sun, S. Zhou, W. Hu, H. Zhang, J. Dong, Z. Jiang, J. Zhao, J. Li, W. Yan, M. Wang, *Nat. Commun.* **2018**, *9*, 2885; b) J. S. Ha, Y. Park, J.-Y. Jeong, S. H. Lee, S. J. Lee, I. T. Kim, S. H. Park, H. Jin, S. M. Kim, S. Choi, C. Kim, S. M. Choi, B. K. Kang, H. M. Lee, Y. S. Park, *Adv. Sci.* **2024**, *11*, 2401782.

[5] J. K. Nørskov, J. Rossmeisl, A. Logadottir, L. Lindqvist, J. R. Kitchin, T. Bligaard, H. Jónsson, *J. Phys. Chem. B* **2004**, *108*, 17886.

[6] Y. Li, M.-C. Kim, C. Xia, W. T. Hong, J. Kim, G. Bae, Y. S. Jang, S. Y. Jeong, E. Sim, C. H. Choi, T.-H. Kim, K. H. Kim, J. K. Kim, *Appl. Catal. B* **2024**, *343*, 123516.

[7] S. Han, Y. Chen, Y. Hao, Y. Xie, D. Xie, Y. Chen, Y. Xiong, Z. He, F. Hu, L. Li, J. Zhu, S. Peng, *Sci. China Mater.* **2021**, *64*, 1127.

[8] J. Chen, Q. Long, K. Xiao, T. Ouyang, N. Li, S. Ye, Z.-Q. Liu, *Sci. Bull.* **2021**, *66*, 1063.

[9] M. Yu, S. Zhou, Z. Wang, J. Zhao, J. Qiu, *Nano Energy* **2018**, *44*, 181.

[10] L. Zhao, B. Dong, S. Li, L. Zhou, L. Lai, Z. Wang, S. Zhao, M. Han, K. Gao, M. Lu, X. Xie, B. Chen, Z. Liu, X. Wang, H. Zhang, H. Li, J. Liu, H. Zhang, X. Huang, W. Huang, *ACS Nano* **2017**, *11*, 5800.

[11] L. Yan, B. Zhang, S. Wu, J. Yu, *J. Mater. Chem. A* **2020**, *8*, 14234.

[12] D. Guo, X. Li, Y. Jiao, H. Yan, A. Wu, G. Yang, Y. Wang, C. Tian, H. Fu, *Nano Res.* **2022**, *15*, 238.

[13] L. Xiu, Z. Wang, M. Yu, X. Wu, J. Qiu, *ACS Nano* **2018**, *12*, 8017.

[14] N. Hao, Y. Wei, J. Wang, Z. Wang, Z. Zhu, S. Zhao, M. Han, X. Huang, *RSC Advances* **2018**, *8*, 20576.

[15] C. Wang, X.-D. Zhu, Y.-C. Mao, F. Wang, X.-T. Gao, S.-Y. Qiu, S.-R. Le, K.-N. Sun, *Chem. Commun.* **2019**, *55*, 1237.

[16] C. Hao, Y. Wu, Y. An, B. Cui, J. Lin, X. Li, D. Wang, M. Jiang, Z. Cheng, S. Hu, *Mater. Today Energy* **2019**, *12*, 453.

[17] N. C. S. Selvam, J. Lee, G. H. Choi, M. J. Oh, S. Xu, B. Lim, P. J. Yoo, *J. Mater. Chem. A* **2019**, *7*, 27383.

[18] N. Li, J. Han, K. Yao, M. Han, Z. Wang, Y. Liu, L. Liu, H. Liang, *J. Mater. Sci. Technol.* **2022**, *106*, 90.

[19] C.-F. Du, X. Sun, H. Yu, W. Fang, Y. Jing, Y. Wang, S. Li, X. Liu, Q. Yan, *Infomat* **2020**, *2*, 950.

[20] M. Tian, Y. Jiang, H. Tong, Y. Xu, L. Xia, *ChemNanoMat* **2020**, *6*, 154.

[21] Y. Liu, L. Bai, T. Li, H. Liu, X. Wang, L. Zhang, X. Hao, C. He, S. Guo, *Mater. Adv.* **2022**, *3*, 4359.

[22] H. Zou, B. He, P. Kuang, J. Yu, K. Fan, *ACS Appl. Mater. Inter.* **2018**, *10*, 22311.

[23] J. Kwon, H. Han, S. Jo, S. Choi, K. Y. Chung, G. Ali, K. Park, U. Paik, T. Song, *Adv. Energy Mater.* **2021**, *11*, 2100624.

[24] Y. Shi, Y. Yu, Y. Liang, Y. Du, B. Zhang, *Angew. Chem. Int. Ed.* **2019**, *58*, 3769.

[25] Z. Kuang, S. Liu, X. Li, M. Wang, X. Ren, J. Ding, R. Ge, W. Zhou, A. I. Rykov, M. T. Sougrati, P.-E. Lippens, Y. Huang, J. Wang, *J. Energy Chem.* **2021**, *57*, 212.

[26] Z. Zeng, L. Y. Gan, H. Bin Yang, X. Su, J. Gao, W. Liu, H. Matsumoto, J. Gong, J. Zhang, W. Cai, Z. Zhang, Y. Yan, B. Liu, P. Chen, *Nat. Commun.* **2021**, *12*, 4088.

[27] L. Lv, Z. Li, K.-H. Xue, Y. Ruan, X. Ao, H. Wan, X. Miao, B. Zhang, J. Jiang, C. Wang, K. Ostrikov, *Nano Energy* **2018**, *47*, 275.

[28] Q. Wu, S. Wang, J. Guo, X. Feng, H. Li, S. Lv, Y. Zhou, Z. Chen, *Nano Res.* **2022**, *15*, 1901.

[29] F. Zhou, X. Zhang, R. Sa, S. Zhang, Z. Wen, R. Wang, *Chem. Eng. J.* **2020**, *397*, 125454.

[30] M. Yu, Z. Wang, J. Liu, F. Sun, P. Yang, J. Qiu, *Nano Energy* **2019**, *63*, 103880.

[31] H. Yang, C. Dong, H. Wang, R. Qi, L. Gong, Y. Lu, C. He, S. Chen, B. You, H. Liu, J. Yao, X. Jiang, X. Guo, B. Y. Xia, *Proc. Natl. Acad. Sci. U.S.A.* **2022**, *119*, e2202812119.

[32] S. Li, L. Wang, H. Su, A. N. Hong, Y. Wang, H. Yang, L. Ge, W. Song, J. Liu, T. Ma, X. Bu, P. Feng, *Adv. Funct. Mater.* **2022**, *32*, 2200733.

[33] Q. Yan, P. Yan, T. Wei, G. Wang, K. Cheng, K. Ye, K. Zhu, J. Yan, D. Cao, Y. Li, *J. Mater. Chem. A* **2019**, *7*, 2831.

[34] J. Dong, Y. Wang, Q. Jiang, Z.-A. Nan, F. R. Fan, Z.-Q. Tian, *J. Mater. Chem. A* **2021**, *9*, 20058.

[35] D. Lim, C. Lim, M. Hwang, M. Kim, S. E. Shim, S.-H. Baeck, *J. Power Sources* **2021**, *490*, 229552.

[36] Y. Wang, Y. Zhou, M. Han, Y. Xi, H. You, X. Hao, Z. Li, J. Zhou, D. Song, D. Wang, F. Gao, *Small* **2019**, *15*, 1805435.

[37] D. Zhou, X. Xiong, Z. Cai, N. Han, Y. Jia, Q. Xie, X. Duan, T. Xie, X. Zheng, X. Sun, X. Duan, *Small Methods* **2018**, *2*, 1800083.

[38] G. Yilmaz, C. F. Tan, Y.-F. Lim, G. W. Ho, *Adv. Energy Mater.* **2019**, *9*, 1802983.

[39] E. López-Fernández, C. Gómez-Sacedón, J. Gil-Rostra, J. P. Espinós, A. R. González-Elipe, F. Yubero, A. de Lucas-Consuegra, *Chem. Eng. J.* **2022**, *433*, 133774.

[40] S. Yin, W. Tu, Y. Sheng, Y. Du, M. Kraft, A. Borgna, R. Xu, *Adv. Mater.* **2018**, *30*, 1705106.

[41] M. Zha, C. G. Pei, Q. Wang, G. Z. Hu, L. G. Feng, *J. Energy Chem.* **2020**, *47*, 166.
